# Multi-locus GWAS analysis identifies genomic regions associated with resistance to ergot (*Claviceps africana*) in sorghum

**DOI:** 10.1371/journal.pone.0325224

**Published:** 2025-06-23

**Authors:** Dejene Kebede, Patrick Rubaihayo, Geoffrey Tusiime, Arfang Badji, Thomas Odong, Mildred Ochwo-Ssemakula, Richard Edema, Paul Gibson, Isaac Onziga Dramadri

**Affiliations:** 1 Department of Crop Science and Horticulture, College of Agricultural and Environmental Sciences, Makerere University, Kampala, Uganda; 2 Makerere University Regional Centre for Crop Improvement, College of Agricultural and Environmental Sciences, Makerere University, Kampala, Uganda; 3 Ethiopian Institute of Agricultural Research, Addis Ababa, Ethiopia; 4 Regional Universities Forum for Capacity Building in Agriculture (RUFORUM), Kampala, Uganda; 5 Department of Soil Science and Land Use Management, College of Agricultural and Environmental Sciences, Makerere University, Kampala, Uganda; Jeju National University, KOREA, REPUBLIC OF

## Abstract

Deployment of resistant genotypes is one of the major components of ergot disease management in sorghum. Identification of genomic regions and candidate genes associated with resistance to ergot is a key step to facilitate sorghum breeding for resistance to ergot. The objective of this study was to identify genomic regions associated with resistance to ergot in sorghum. A total of 330 lines from the global sorghum association panel (SAP) population genotyped with 114920 genome wide SNP markers were used in this study. The SAP was evaluated for resistance to ergot in two field trials conducted at MUARIK during the first and second seasons of 2020 and 2021, respectively. Six multi-locus genome wide association studies (ML – GWAS) methods were used to identify significant quantitative trait nucleotides (QTNs). ML – GWAS analysis using SAP population detected thirty-eight significant QTNs. Further analysis identified 19 QTNs with relatively higher phenotypic effects ranging from 5–12.7%. Additionally, 47 candidate genes linked with the significant QTNs were detected. Most of the identified genes were involved in several biological processes including DNA and RNA binding, metal ion binding, regulation of transcription and translation and transduction signaling related to defense response against pathogen infections. This study contributes to the identification of significant QTNs and candidate genes associated with resistance to ergot in sorghum.

## Introduction

Sorghum (*Sorghum bicolor* (L) Moench) is one of the most important crops in the world, which is used widely as livestock feed, brewing, food, and biofuels [1]. Sorghum is an important source of food in developing countries, in Africa and Asia more than 95% of sorghum produce is used for human consumption mainly as food or as a raw- material for industries [2]. Nutritionally sorghum grain is equal or superior to other staple cereals since it is a useful source of quality protein and various minerals [3]. Important food uses of sorghum in different countries include: injera (a thin bread) (Ethiopia); tortilla (Latin America); porridges (west Africa); unleavened breads such as roti (India); chapatti (south Asia); bogobe (Botswana), sankati (southern Africa), ogi (Nigeria) and a variety of traditional foods such as fermented and non-fermented porridge and semi-leavened bread in Uganda [4,5,6]. It is also used for making a variety of drinks such as fermented beverages [4].

Sorghum is a preferred crop in sub-Saharan African countries because of its ability to withstand harsh environmental conditions compared to other cereal crops such as Maize and Wheat, but its production is constrained by arrays of abiotic and biotic factors including ergot or sugary (C*laviceps species*) disease [7]. Ergot is a threat to sorghum production as it causes a significant yield loss worldwide because of its occurrence almost in all continents, especially in regions with high humid and wet conditions [6,8]. Ergot is caused by three *Claviceps species* namely, *Claviceps africana, Claviceps sorghi and Claviceps sorghicola.* The *Claviceps sorghi* was first described and endemic to India, *Claviceps africana and Claviceps sorghicola* are endemic to Africa and Japan, respectively. Ergot caused by *Claviceps africana* is the most prevalent among the three Claviceps species and distributed worldwide [9]. Ergot in Africa which is caused by *Claviceps africana* was first recognized as a potential threat to F1 hybrid seed production in the l960s [10]. In eastern and southern Africa especially in Zimbabwe and South Africa, the disease was a problem amongst the male sterile lines used in hybrid breeding programs [9]. In the presence of the ergot pathogen and when weather conditions are favorable for ergot infection, a reduction in pollen production from male-fertile lines can result in a serious yield loss [11].

Reports from southern Africa, Mexico and the United States indicated that a yield loss of up to 50% and 80% were observed in sorghum grain and hybrid seed production, respectively [12,13]. Ergot contaminated grain can reduce feed intake and cause toxicity when fed to livestock [14]. Infected seeds have lower germination and seedling emergence and can also increase incidence and severity of grain molds such as *cerebella* species, *Curvularia* species, *Fusarium* species, *Alternaria* species, and *Cladosporium* species [15] hence development of resistant genotypes is an important task to reduce ergot infestations. Due to the quantitative inheritance of sorghum traits that influence resistance to ergot, conventional breeding methods for improving sorghum resistance to ergot is slow [8]. Therefore, integration of molecular and conventional approaches should improve efficiency and precision of selection. For effective employment of molecular breeding techniques such as marker assisted selection in sorghum improvement, identification of genomic regions responsible for resistance and determining their molecular and biological functions is a pre-request [14]. In previous study, Parh et al. [14] carried out a QTL mapping and analysis of sorghum recombinant inbred lines population R931945-2–2 × IS 8525 using three hundred and three markers including thirty-six simple sequence repeat, one hundred and seventeen amplified fragment length polymorphism, one hundred and forty-eight diversity arrays technology markers and two morphological traits. They identified nine, five, and four QTLs linked to resistance to ergot traits: percentage ergot infection, pollen quantity, and pollen viability, respectively using composite interval mapping. However, identification of genomic regions based on bi – parental populations such as RIL and F2 result in limited genomic resolutions and restricted allelic diversity as only allelic segregates between and among the parents of the recombinant progenies can be assayed [16].

Genome-wide association studies (GWAS) have become an important tool for identification of genomic regions associated with quantitatively inherited traits. Two GWAS approaches including single-locus GWAS and multi – locus GWAS approaches have been employed in detection of important genomic regions; significant QTNs and putative candidate genes [17]. Single locus GWAS methods such as efficient mixed-model association eXpedited (EMMAX), mixed linear model (MLM) and factored spectrally transformed linear mixed models (FaST-LMM) are among the extensively used approaches [18]. However, single locus GWAS methods are limited in detecting marginal effects SNPs influenced by the polygenic background and require multiple critical-value test corrections such as Bonferroni to reduce false-positive rates [19]. To address the shortcomings of single locus GWAS, the multi-locus GWAS approach has been developed as a multi-dimensional genome scan method in which the effects of all markers are estimated simultaneously [20]. Six multi locus GWAS methods including mrMLM (multi-locus random-SNP-effect MLM) [21], FASTmrMLM (fast mrMLM) [22], ISIS EM-BLASSO (iterative modified-sure independence screening expectation-maximization-Bayesian least absolute shrinkage and selection operator) [23], pKWmEB (integration of Kruskal-Wallis test with empirical Bayes) [24], FASTmrEMMA (fast multi-locus random-SNP-effect efficient mixed model analysis) [25], and pLARmEB (polygenic-background-control-based least angle regression plus empirical Bayes) [26] have been used to identify significant QTNs associated with important traits. Multi locus GWAS methods have been reported to have lower false-positive rates and high power and accuracy in identifying significant SNPs [18]. The present study was conducted to identify genomic regions and candidate genes associated with resistance to ergot in sorghum through multi – locus GWAS methods using a sorghum association panel.

## Methodology

### Phenotyping

#### Experimental site.

The experiment was conducted at Makerere University Agricultural Research Institute at Kabanyolo (MUARIK), Uganda during the first season (April – May) of 2020 and the second season (September – December) of 2021. MUARIK is located at 0°28’N and 32°37’E with an altitude of 1200 meter above sea level and deep ferrallitic soils with a pH range of 5.2 to 6.0 [27].

#### Plant materials.

The experiment constituted a total of 330 sorghum genotypes from the sorghum association panel (SAP). The SAP included accessions from all major cultivated races and geographic centers of diversity, including 31 genotypes from Sudan, 30 from Ethiopia, 19 from India, 15 from Nigeria, 14 from Uganda, 4 from Japan, 167 unknown sources (S1 Table) [28].

#### Experimental design and field layout.

The experiment was planted in an alpha-lattice design with two replications. Each genotype was planted on a plot area consisting of two rows with a plot length of 5 m. A spacing of 1.5 m and 2 m between plots and replications were used, respectively. A spacing of 0.75 m between rows and 0.15 m within rows was used, and fields were weeded three times.

#### Data collection and analysis.

In 2020, planting was conducted on 31 August, and data on ergot resistance traits were collected in December for three consecutive weeks: 14 weeks after planting, 15 weeks after planting, and the final score was collected 16 weeks after planting. Likewise, in 2021, planting was carried out in April, and data on ergot infection was recorded in July for three consecutive weeks (14WAP, 15WAP, and 16WAP). Ergot severity (ES) was collected based on 1–5 scale of visual scoring as described by Musabyimana et al. [29] (Table 1).

**Table 1:** Ergot severity score and disease reactions.

Score	Infection (%)	Disease reaction
1	No infection	Highly resistant
2	1–10% infection	Resistant
3	11-25% infection	Moderately resistant
4	26-50% infection	Susceptible
5	>50% infection	Highly susceptible

Ergot incidence (EI) was estimated by counting all the individual plants with ergot disease symptoms in each plot and computed using the following formula.


$$\begin{array}{c} \text{Yijk}=\mu +\text{Gi}+\text{Bj/Yk}+\text{Yk\textbackslash{} + GiYk}+\epsilon \text{ijk} \end{array}$$

Pollen quantity was estimated following the procedures used by Parh et al. [30] when 50% of plants flowers in each plot, five heads per plot were flicked once and rated 1 to 10 (1 for no visible pollen, and 10 for ample quantity of visible pollen) by observing the density of the resultant clouds of pollen. Days to 50% flowering was recorded as the number of days from planting to when 50% of plants shaded pollen [31].

Statistical analysis of phenotypic data was performed using R statistical package for Windows V- 4.1.1. Agricolae, ggplot2 and corplot packages in R – software were used to determine coefficient of variations, standard error, standard deviations, heritability and Pearson’s correlation analysis [32].

Analysis of the phenotype data was performed using the model:


$$\begin{array}{c} \text{Yijk}=\mu +\text{Gi}+\text{Bj/Yk}+\text{Yk\textbackslash{} + GiYk}+\epsilon \text{ijk} \end{array}$$

Where, Yijk is the response of the i^th^ genotype within the j^th^ replication of the k^th^ year. μ = grand mean, Gi = the effect of the i^th^ genotype, Yk = the effect of the k^th^ year, Bj/Yk = the effect of j^th^ replication nested within k^th^ year, GiYk = the interaction effect of the i^th^ genotype and the k^th^ year, and Єijk = the residual error.

### Genotyping

DNA extraction was done using DNeasy Plant Mini Kit (QIAGEN) as described in Morris et al. [28]. Genotyping was carried out using multiplexed (96- or 384-plex) genotyping-by-sequencing with ApeKI restriction enzyme (recognition site: G|CWCG) on an Illumina HiSeq/Genome Analyzer IIx. Sequences were mapped to the BTx623 sorghum reference genome [1] using BWA tool and SNPs were called using the TASSEL 3.0 GBS pipeline as cited in Morris et al. [28]. Tags, unique sequence of 64 bp length that included a leading 4 bp C[T/A]GC signature from the cut site, were identified and tags with at least 10X coverage were retained. Genotype data filtering to identify polymorphic, non – redundant SNPs with less missing data (<20%) and > 5% minor allele frequency (MAF) was done using TASSEL 5.0 software [33] and retained a total of 114,920 SNPs for the GWAS analysis.

### Genome wide association analysis

Association analysis using genome-wide SNP markers for resistance to ergot in sorghum was performed using six mrMLM.GUI (multi-locus random-SNP-effect Mixed Linear Model with Graphical User Interface) methods, including mrMLM, FASTmrMLM, FASTmrEMMA, pLARmEB, pKWmEB and ISIS EM-BLASSO in R – software within the mrMLM package [19]. All the six methods used the population structure and kinship matrices. Population structure was determined using principal component analysis (PCA) in Tassel Version – 5 software [33]. The mrMLM.GUI software was allowed to calculate kinship internally. QTNs with a LOD score greater than 3.0 were considered significant for all the six mrMLM methods [25]. QTNs detected by at least three ML – GWAS methods were considered reliable [19]. The resulting −log10 (P) values from the ML-GWAS methods were used to draw the Manhattan and Q–Q plots using the mrMLM. GUI package [19].

### Population structure analysis

To determine the population structure, STRUCTURE v2.3.4 software [34] was used. Run length was given as 10,000 burning period length followed by 10,000 Markov Chain Monte Carlo (MCMC) replications. Each ancestry kinship (K) value was run for 5 replications with K value varying from 1 to 10. The optimum K value was predicted by calculating ΔK according to the Evanno method [35] using Structure Harvester [36].

### Candidate gene analysis

Candidate genes associated with significant QTNs were searched within the range of 50 kb linkage disequilibrium (LD) decay (28). Identification and annotation of the candidate genes were performed using the Sorghum BTx623 reference genome V-3 available on the Ensemble plants browser. Functional annotation of the candidate gene was based on Ensemble Plants and Gramene annotation [37].

## Results

### Phenotypic variations

Significant variability was observed among genotypes for ergot incidence, severity, hundred seed weight, pollen quantity and days to 50% flowering (Table 2). The average disease incidence score ranged between 3–100% at 14WAP and 4–100% at 15WAP and 16WAP with overall mean incidence of 45.36, 60.06 and 67.39% at 14WAP, 15WAP and 16WAP, respectively (Table 2). Ergot severity varied between 1–5 at 14WAP and 15WAP and 1.5–5 at 16WAP. The mean ergot severity was 2.51, 2.94 and 3.02 at 14WAP, 15WAP and 16WAP, respectively. Days to 50% flowering and pollen quantity varied between 56–108 days and 5.0–9.0, respectively. Coefficient of variations ranged between 6.3–40.9% across all recorded traits. Pollen quantity (6.3) and days to 50% flowering (6.82) had the lowest coefficient of variations, while ergot incidence had the highest coefficient of variations at 14WAP (40.98%), 15WAP (30.02%) and 16WAP (25.1%). Standard error of means ranged between 0.03–1.52 with pollen quantity (0.03) and ergot incidence at 14WAP (1.52) recorded the lowest and highest standard error of means, respectively. Broad sense heritability for all recorded traits varied between 32.2–85.04%. The highest heritability was observed for ergot severity at 15WAP, while the lowest heritability was recorded for pollen quantity (Table 2).

**Table 2:** Phenotypic variability of ergot incidence, severity and other traits combined across years.

Traits	Minimum	Maximum	Mean	SD	SE	CV	H (%)
EI – 14WAP	3.0	100.0	45.36	26.54	1.52	40.98	66.0
EI – 15WAP	4.0	100.0	60.06	25.95	1.47	30.02	72.0
EI – 16WAP	4.0	100.0	67.39	25.21	1.42	25.10	75.0
ES – 14WAP	1.0	5.0	2.51	0.80	0.05	16.67	77.0
ES – 15WAP	1.0	5.0	2.94	0.71	0.04	13.32	85.0
ES – 16WAP	1.5	5.0	3.02	0.76	0.04	14.28	84.0
DTF	56.0	108.0	80.7	8.72	0.49	6.82	63.0
PQ	5.0	9.0	6.76	0.58	0.03	6.29	32.0

EI – ergot incidence, ES – ergot severity, WAP – weeks after planting, DTF – days to 50% flowering, PQ – pollen quantity, CV – coefficient of variations, SE – standard error, H – heritability in broad sense, SD – standard deviation.

### Genotype by environment interaction

The analysis of variance revealed a highly significant effect for most of the recorded traits among genotypes, years, and genotype by year interactions (Table 3). Highly significant variation was observed among genotypes and years for most of the recorded traits. Significant genotype by year interaction was observed for all traits. Disease incidence and severity score were higher score in 2020 compared to 2021 growing season. In 2020 the mean disease incidence was 34.2% at 14WAP, 60.8% at 15WAP and 69.4% at 16WAP, while severity was 1.9, 2.8 and 2.9 at 14WAP, 15WAP and 16WAP, respectively. Whereas, in 2021, the mean disease incidence was 54.3% at 14WAP, 61.2% at 15WAP and 66.8% at 16WAP, while severity score was 2.9, 3.1 and 3.2 at 14WAP, 15WAP and 16WAP, respectively (Table 3).

**Table 3:** Analysis of variance for ergot incidence, severity, days to 50% flowering and pollen quantity.

SOV	DF	Incidence			Severity			DTF (days)	PQ (1–10)
		14WAP	15WAP	16WAP	14WAP	15WAP	16WAP		
Year	1	103537**	797^ns^	457.2 ^ns^	263**	38.1**	33.3**	9871**	21.4**
Rep/Year	2	5917**	7725**	1861**	1.2**	0.24 ^ns^	1.20**	533**	4.36**
Genotype	329	983**	1217**	1193**	0.76**	1.04**	1.2**	120**	0.42**
Geno * Year		458*	524**	472.9**	0.29**	0.34**	0.39**	69**	0.39**
Error		354	337	297.1	0.18	0.16	0.19	30.3	0.18

*, ** = Significant at P < 0.05 and P < 0.01, respectively, SOV- source of variations, DF – degree of freedom, WAP – weeks after planting, DTF – days to 50% flowering and PQ – pollen quantity.

### Pearson’s correlation between resistance to ergot traits, DTF and PQ

**Fig 1:**
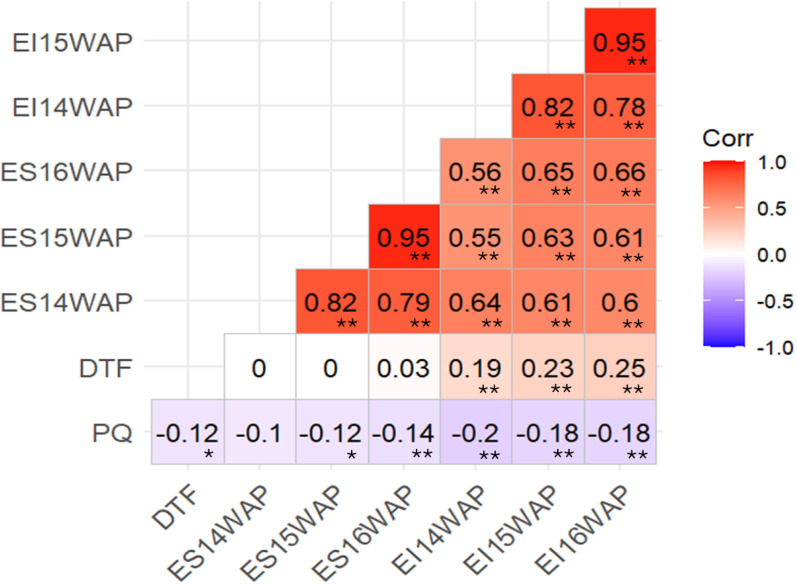
Pearson’s correlation among ergot incidence, severity, DTF and PQ combined across years. *, ** = Significant at P < 0.05 and P < 0.01, respectively, EI – ergot incidence, ES – ergot severity, WAP – weeks after planting, DTF – days to 50% flowering, PQ – pollen quantity and Corr – Pearson’s correlation coefficient.

A highly significant correlation was observed among resistance to ergot traits including EI16WAP, EI15WAP, EI14WAP, ES16WAP, ES15WAP and ES14WAP (Fig 1). PQ exhibited significant negative relationship with EI – 16WAP (r = −0.18, P < 0.01), EI – 15WAP (r = −0.18, P < 0.01), EI – 14WAP (r = −0.2, P < 0.01), ES – 16WAP(r = −0.14, P < 0.01) ES – 15WAP(r = −0.12, P < 0.05) ES – 14WAP(r = −0.1) and DTF (r = −0.12, P < 0.05) (Fig 1). DTF showed significant positive correlation with EI16WAP (r = 0.25, P < 0.01), EI15WAP (r = 0.23, P < 0.01), EI14WAP (r = 0.19, P < 0.01) (Fig 1).

### Population structure analysis

**Fig 2:**
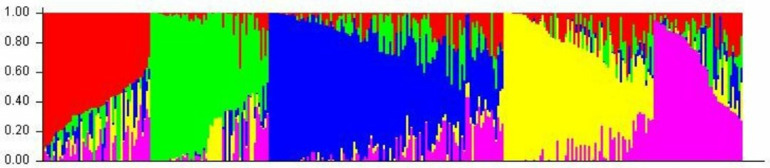
Population structure of SAP populations used for GWAS analysis.

The findings from the population structure analysis are illustrated in Fig 2. The analysis results revealed the presence of five distinct sub-populations within the 330 global sorghum lines that were included in the SAP population utilized for this study. Each of these sub-populations exhibits unique characteristics, highlighting the diversity present within the sorghum SAP population.

### Genome wide association analysis

**Fig 3:**
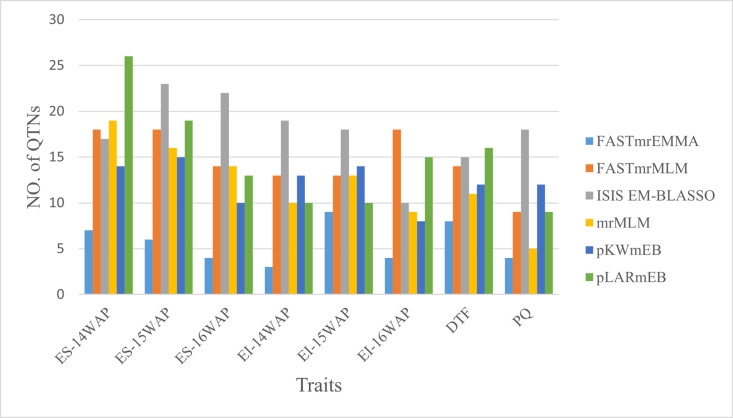
Significant QTNs identified by six different ML – GWAS methods for ergot incidence, severity, and agronomic traits. WAP – weeks after planting, ES – ergot severity, EI – ergot incidence, DTF – days to 50% flowering, PQ – pollen quantity.

Six multi-locus GWAS (ML-GWAS) methods; mrMLM, FASTmrMLM, FASTmrEMMA, and pLARmEB, ISIS EM-BLASSO and pKWmEB were employed to identify significant QTNs associated with ergot incidence and severity at 14WAP, 15WAP and 16WAP, DTF, and PQ (Table 4). Multi-locus models Q–Q plots show consistent with optimal trends, implying that the false-positive errors were controlled well (S1 Fig). A total of 437 significant QTNs were detected by six ML – GWAS methods for all traits. These include ergot incidence at 14WAP (54 QTNs), 15WAP (51) and 16WAP (52), severity at 14WAP (76), 15WAP (60) and 16WAP (52), days to 50% flowering (48) and pollen quantity (44) (Table 4). The ISIS EM-BLASSO method detected the highest number of QTNs of all the six ML – GWAS methods, as 47, 62, 15 and 18 QTNs were detected for ergot incidence (14WAP, 15WAP and 16WAP), severity (14WAP, 15WAP and 16WAP), DTF and PQ, respectively. The least number of QTNs were detected by FASTmrEMMA with a total of 16, 17, 8 and 4 QTNs were detected for ergot incidence (14WAP, 15WAP and 16WAP), severity (14WAP, 15WAP and 16WAP), DTF and PQ, respectively (Fig 3). FASTmrMLM detected 44 QTNs for ergot incidence (14WAP, 15WAP and 16WAP), 50 QTNs for ergot severity (14WAP, 15WAP and 16WAP), 14 QTNs for DTF and 9 QTNs for PQ. In pLARmEB a total of 58, 35, 16 and 9 QTNs were detected for ergot severity (14WAP, 15WAP and 16WAP), incidence (14WAP, 15WAP and 16WAP), DTF and PQ, respectively. The pKWmEB detected thirty-five for ergot incidence (14WAP, 15WAP and 16WAP), 39 for ergot severity (14WAP, 15WAP and 16WAP), 12 for DTF and 12 QTNs for PQ. Furthermore, mrMLM detected a total of 49, 32, 11 and 5 QTNs for ergot severity (14WAP, 15WAP and 16WAP), ergot incidence (14WAP, 15WAP and 16WAP), DTF and PQ, respectively (Fig 3).

**Table 4:** Total number of QTNs identified by six ML – GWAS methods for different traits.

Trait	No. of QTNs	QTNs effects		LOD score		r^2^ (%)	
		Min	Max	Min	Max	Min	Max
EI – 14WAP	54	−13.77	8.21	3.03	11.53	0.65	12.79
EI – 15WAP	51	−12.24	11.63	3.03	10.54	0.97	9.02
EI – 16WAP	52	−13.94	13.82	3.04	11.87	1.12	13.99
ES – 14WAP	76	−0.23	0.29	3.01	12.47	0.00	12.70
ES – 15WAP	60	−0.24	0.33	3.06	19.21	0.00	11.64
ES – 16WAP	52	−0.28	0.45	3.02	10.72	0.00	11.72
DTF	48	−5.73	3.46	3.09	9.05	1.10	11.78
PQ	44	−0.22	0.17	3.08	8.57	0.00	9.18

EI – ergot incidence, ES – ergot severity, WAP – weeks after planting, DTF – days to 50% flowering, PQ – pollen quantity, QTNs, quantitative trait nucleotide, LOD – logarithms of odds, Min – minimum and Max – maximum.

### QTNs detected by at least three ML – GWAS methods

**Fig 4:**
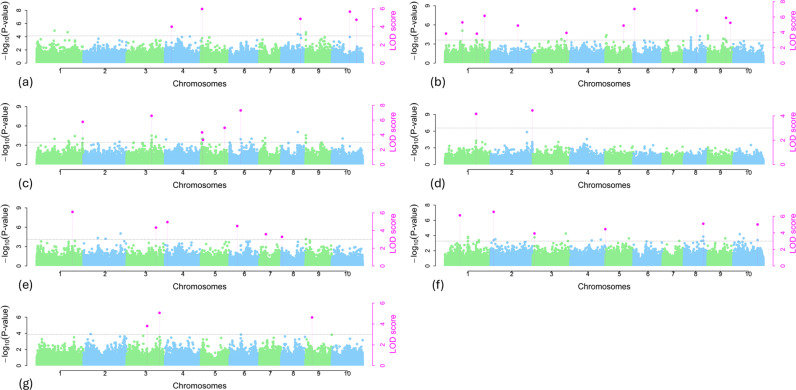
Manhattan plot showing p-values and genome-wide associations for resistance to ergot and other traits (a) ES-14WAP, (b) ES-15WAP, (c) ES-16WAP, (d) EI-14WAP, (e) EI-15WAP, (f) DTF and (g) PQ. Each marker median of the − log10(p) values from the multi-locus random- QTN -effect mixed linear model (mrMLM), factored spectrally transformed multi-locus random- QTN -effect MLM (FASTmrMLM), and factored spectrally transformed multi-locus random- QTN -effect efficient mixed-model association (FASTmrEMMA) approaches were used to draw the Manhattan. The dots are indicated by light colors QTNs; all QTNs commonly identified by three approaches are indicated by pink dots with dotted vertical lines.

Thirty-eight significant QTNs were identified by at least three methods associated with ergot incidence, severity, DTF and PQ, with one QTN (S5_1649273) identified in both ES-14WAP and ES-16WAP (S2 Table). QTN: S4_3127636 linked with EI – 14WAP was detected by all the six ML – GWAS methods. Three QTNs including, S9_51757008 (ES – 15WAP), S1_72403757 (ES – 16WAP), and S2_3639089 (DTF) were detected simultaneously by five different methods. The other 12 and 22 QTNs were detected by four and three different ML – GWAS methods, respectively (S2 Table). The QTNs associated with ergot incidence (EI – 14WAP, EI – 15WAP and EI – 16WAP) were detected on chromosomes 1, 2, 3, 4, 6, 7, and 8, while the QTNs associated with ergot severity (ES – 14WAP, ES – 15WAP and ES – 16WAP) were detected on all chromosomes except chromosome 7 and most of the QTNs are located on chromosome 1 and 5 with 5 QTNs in each chromosome. QTNs associated with DTF were detected on chromosomes 1, 2, 3, 4, 8 and 10. Furthermore, QTNs associated with PQ were detected on chromosomes 3 and 9 (Fig 4).

Of the thirty-eight significant QTNs, 19 QTNs had greater than 5% effects on the phenotypic variance of evaluated traits (Table 5). Four QTNs associated with ergot incidence (1 with EI – 14WAP, and 3 with EI – 15WAP) including, S1_56665164, S4_3127636, S3_64616775 and S7_10036871 explained up to 9.32% of the phenotypic expressions in ergot incidence, with the highest contribution coming from S1_56665164 (2.16–9.32%) and S4_3127636 (2.37–5.63%). The LOD score ranged between 3.38–9.12 and QTNs, S1_56665164 (3.57–9.12) and S4_3127636 (4.45–7.05) had the highest LOD score (Table 5).

**Table 5:** Selected QTNs detected by at least three ML – GWAS methods.

Trait	Method	QTN	Chr	Position (bp)	LOD score	r^2^ (%)	MAF	Alleles
ES –14WAP	1, 4, 5, 6	S10_36991277.	10	36991277	3.27 - 7.44	2.15 - 12.7	0.21	G/A
	1, 2, 4	S5_1649273.	5	1649273	3.34 - 7.37	4.85 - 7.90	0.30	T/C
ES-15WAP	1, 2, 4, 5	S6_1811916.	6	1811916	6.55 - 19.21	4.54 - 11.6	0.47	T/C
	1, 2, 4, 5	S8_40292627.	8	40292627	5.46 - 14.98	5.49 - 10.8	0.23	T/C
	1, 5, 6	S1_26468239.	1	26468239	4.32 - 7.45	2.29 - 5.13	0.10	G/A
	2, 4, 5	S2_62482269.	2	62482269	3.71 - 6.59	3.44 - 7.04	0.17	T/C
ES –16WAP	1, 2, 4, 5, 6	S1_72403757.	1	72403757	4.85 - 10.14	3.76 - 11.4	0.21	T/A
	1, 3, 4, 6	S5_1649273.	5	1649273	3.11 - 4.99	3.17 - 5.95	0.30	T/C
	2, 4, 5, 6	S6_41603668.	6	41603668	4.61 - 10.7	4.90 - 7.44	0.07	A/G
	1, 2, 4	S5_56592572.	5	56592572	3.44 - 6.46	1.84 - 10.2	0.17	C/T
	1, 2, 6	S3_59282235.	3	59282235	3.02 - 8.71	2.14 - 6.66	0.14	G/T
EI – 14WAP	4, 5, 6	S1_56665164.	1	56665164	3.57 - 9.12	2.16 - 9.32	0.46	G/C
EI – 15WAP	1–6	S4_3127636.	4	3127636	4.45 - 7.05	2.37 - 5.63	0.44	C/T
	2, 3, 5	S3_64616775.	3	64616775	3.81 - 6.24	2.40 - 4.97	0.44	C/T
	1, 2, 3	S7_10036871.	7	10036871	3.38 - 3.65	1.74 - 4.88	0.17	A/G
DTF	1, 2, 4, 6	S1_20799733.	1	20799733	5.83 - 8.28	5.95 - 11.3	0.20	C/A
	1, 2, 3, 5	S3_2543818.	3	2543818	3.54 - 6.74	2.85 - 5.97	0.21	A/G
	1, 3, 5	S8_50806664.	8	50806664	4.68 - 9.05	3.33 - 6.29	0.41	C/A
PQ	1, 2, 4	S3_53363038.	3	53363038	3.44 - 4.58	2.30 - 6.52	0.13	A/G
	4, 5, 6	S3_69400934.	3	69400934	3.84 - 8.18	2.91 - 7.66	0.17	G/T

1, mrMLM; 2, FASTmrMLM; 3, FASTmrEMMA; 4, pLARmEB; 5, pKWmEB; 6, ISIS EM-BLASSO, EI – ergot incidence, ES – ergot severity, WAP – weeks after planting, DTF – days to 50% flowering, PQ – pollen quantity, Chr – chromosome, QTNs – quantitative trait nucleotide, MAF – minor allele frequency and LOD – logarithms of odds.

Ten QTNs linked with ergot severity (2 SNP with ES – 14WAP, 4 with ES – 15WAP & 5 with ES – 16WAP) were detected with relatively higher phenotypic effect including S10_36991277, S5_1649273, S6_1811916, S8_40292627, S1_26468239, S2_62482269, S1_72403757, S6_41603668, S5_56592572 and S3_59282235. QTNs S5_1649273 detected simultaneously in ES-14WAP and ES-16WAP. These QTNs explained up to 12.7% of phenotypic variations in disease severity and QTNs, S10_36991277, S6_1811916, S1_72403757, S5_56592572, S8_40292627 had the highest contributions. LOD score for these QTNs varied between 3.02–19.21, with QTNs, S6_1811916 (6.55–19.21), S8_40292627 (5.46–14.98), S1_72403757 (4.85–10.14) and S6_41603668 (4.61–10.7) having the highest LOD score (Table 5).

Three QTNs (S1_20799733, S3_2543818 and S8_50806664) were identified associated with DTF These QTNs explained up to 11.31% of phenotypic variations in DTF. The LOD score of these QTNs varied between 3.54–9.05. For PQ QTNs S3_53363038 and S3_69400934 explained up to 7.66% of the phenotypic variations in PQ and had LOD score ranging from 3.44–8.18 (Table 5).

### Prediction of candidate genes

A total of 109 candidate genes linked with the 19 QTNs were detected for ergot incidence, severity, DTF and PQ. Among the genes identified, 62 genes were uncharacterized or unknown. The other 47 genes had protein descriptions with molecular functions and biological processes (Table 6). Using gene annotation, these candidate genes were categorized into two functional groups related to molecular and biological functions. In the molecular function category, the candidate genes are predicted to have functions related to DNA binding, nucleic acid binding, zinc ion binding, ATP binding, flavin adenine dinucleotide binding, rRNA binding, magnesium ion binding and metal ion binding. In the biological processes category, the candidate genes were predicted to have functions related to regulation of transcription, intracellular signal transduction, negative regulation of DNA endoreduplication, carbohydrate metabolic process, galactose metabolic process, oxidoreductase activity, protein serine/threonine kinase activity, negative regulation of catalytic activity, nucleobase-containing compound metabolic process, fatty acid biosynthetic process, cell communication, macromolecule localization, flower development and defense response (Table 6).

**Table 6:** List of potential candidate genes and their molecular and biological function associated with ergot incidence, severity, DTF and PQ.

Traits	QTNs	Candidate genes	Protein	Molecular function	Biological function
**ES −14 WAP**	S5_1649273.	SORBI_3005G017801	GST N-terminal domain-containing protein	glutathione transferase activity	glutathione transferase activity
		SORBI_3005G018200	Apyrase	nucleoside diphosphate phosphatase activity	ATP binding
		SORBI_3005G018400	NB-ARC domain-containing protein	defense response	defense response
		SORBI_3005G018500	NAC69	DNA binding	regulation of DNA-templated transcription
		SORBI_3005G018900	Phosphoinositide phospholipase C, EC:3.1.4.11	phosphatidylinositol phospholipase C activity	intracellular signal transduction
**ES – 15WAP**	S6_1811916.	SORBI_3006G012200	Flavin-containing monooxygenase	flavin adenine dinucleotide binding indole-3-pyruvate monooxygenase activity, NADP binding	
		SORBI_3006G012300	3’-5’ exonuclease	3’-5’ exonuclease activity and nucleic acid binding	nucleobase-containing compound metabolic process
		SORBI_3006G012500	Protein transport protein Sec24-like	SNARE binding and zinc ion binding	male gamete generation, negative regulation of DNA endoreduplication, pollen development, regulation of cell size
	S1_26468239.	SORBI_3001G247700	3-ketoacyl-CoA synthase	acyltransferase activity, transferring groups other than amino-acyl groups	fatty acid biosynthetic process
		SORBI_3001G247900	GH18 domain-containing protein	hydrolase activity, hydrolyzing O-glycosyl compounds	carbohydrate metabolic process
	S2_62482269.	SORBI_3002G233600	Glycosyl hydrolase family 13 catalytic domain-containing protein	hydrolase activity, hydrolyzing O-glycosyl compounds	carbohydrate metabolic process
		SORBI_3002G233700	Glutamate/malate translocator	chloroplast inner membrane	malate transmembrane transporter activity
		SORBI_3002G233800	Homeobox-leucine zipper protein	DNA-binding transcription factor activity, RNA polymerase II-specific	positive regulation of DNA-templated transcription
		SORBI_3002G233900	Protein SCAI	transcription corepressor activity	DNA-templated transcription
		SORBI_3002G234100	cysteine dioxygenase	metal ion binding	detection of hypoxia
		SORBI_3002G234200	Peroxidase	heme binding and lactoperoxidase activity and metal ion binding	hydrogen peroxide catabolic process response to oxidative stress
		SORBI_3002G234300	SAM-dependent MTase RsmB/NOP-type domain-containing protein	methyltransferase activity and tRNA binding	RNA methylation and tRNA methylation
**ES - 16WAP**	S6_41603668.	SORBI_3006G061500	Ubiquitin-like protease family profile domain-containing protein	deSUMOylase activity	protein desumoylation
	S5_56592572.	SORBI_3005G130051	CCHC-type domain-containing protein	mRNA binding and translation regulator activity	zinc ion binding
		SORBI_3005G130100	Terpene synthase	magnesium ion binding	diterpenoid biosynthetic process
	S3_59282235.	SORBI_3003G253600	Protein kinase domain-containing protein	ATP binding and protein serine/threonine kinase activity	cell communication
		SORBI_3003G254000	Pentacotripeptide-repeat region of PRORP domain-containing protein	mRNA binding	mRNA binding
		SORBI_3003G254100	Protein kinase domain-containing protein	ATP binding	protein kinase activity
		SORBI_3003G254300	Glutaredoxin-dependent peroxiredoxin, EC:1.11.1.25	thioredoxin peroxidase activity	cell redox homeostasis hydrogen peroxide catabolic process
		SORBI_3003G254700	Glycosyltransferases, EC:2.4.-.-	galactosylgalactosylxylosylprotein 3-beta-glucuronosyltransferase activity and xylosyltransferase activity	glucuronoxylan biosynthetic process plant-type secondary cell wall biogenesis
		SORBI_3003G254850	NAC domain-containing protein	DNA binding	regulation of DNA-templated transcription
		SORBI_3003G254900	NAC domain-containing protein	DNA binding	regulation of DNA-templated transcription
		SORBI_3003G255000	NAC domain-containing protein	DNA binding	regulation of DNA-templated transcription
**EI – 14WAP**	S1_56665164.	SORBI_3001G289800	BAH domain-containing protein	chromatin binding	
		SORBI_3001G289900	AT-hook motif nuclear-localized protein	DNA binding	
**EI – 15WAP**	S4_3127636.	SORBI_3004G037900	L-arabinokinase	ATP binding and galactokinase activity	galactose metabolic process
		SORBI_3004G038100	Nuclear pore complex protein NUP160 helical domain-containing protein	structural constituent of nuclear pore	macromolecule localization
		SORBI_3004G038300	Terpene cyclase/mutase family member, EC:5.4.99.-	intramolecular transferase activity	triterpenoid biosynthetic process
		SORBI_3004G038400	Cathepsin propeptide inhibitor domain-containing protein	cysteine-type endopeptidase activity	proteolysis involved in protein catabolic process
	S1_61119088.	SORBI_3001G323801	PMEI domain-containing protein	enzyme inhibitor activity	negative regulation of catalytic activity
	S3_64616775.	SORBI_3003G318100	LysM domain-containing protein	chitin binding	
		SORBI_3003G318200	LysM domain-containing protein	chitin binding	
**DTF**	S1_20799733.	SORBI_3001G221200	Long-chain-alcohol oxidase, EC:1.1.3.20	flavin adenine dinucleotide binding	long-chain-alcohol oxidase activity
		SORBI_3001G221300	Long-chain-alcohol oxidase, EC:1.1.3.20	flavin adenine dinucleotide binding	long-chain-alcohol oxidase activity
	S8_50806664.	SORBI_3008G109300	Fe2OG dioxygenase domain-containing protein	metal ion binding	oxidoreductase activity
		SORBI_3008G109400	Fe2OG dioxygenase domain-containing protein	metal ion binding	oxidoreductase activity
	S3_2543818.	SORBI_3003G028600	phosphoribosylaminoimidazole carboxylase, EC:4.1.1.21, AIR carboxylase	ATP binding and metal ion binding	‘de novo’ IMP biosynthetic process
		SORBI_3003G029100	FRIGIDA-like protein		flower development
**PQ**	S3_53363038.	SORBI_3003G203400	Protein kinase domain-containing protein	ATP binding	protein serine/threonine kinase activity
		SORBI_3003G203500	Aldehyde dehydrogenase domain-containing	aldehyde dehydrogenase (NAD+) activity	
		SORBI_3003G203800	TF-B3 domain-containing protein	DNA binding	
	S3_69400934.	SORBI_3003G380100	DYW_deaminase domain-containing protein	zinc ion binding	

WAP – Weeks after planting, EI – Ergot incidence, ES – Ergot severity, DTF – days to 50% flowering and PQ – pollen quantity.

## Discussions

Ergot is a serious problem particularly in hybrid sorghum production that utilizes male-sterile parents [8]. However, when weather conditions are favorable for infection, all types of sorghum germplasm, fertile or male sterile are susceptible to ergot [38]. Identification of genomic regions associated with resistance to ergot would assist the development of sorghum cultivars resistant to ergot. This study was conducted to identify genomic regions: significant QTNs and candidate genes associated with resistance to ergot in sorghum through multi locus GWAS method using sorghum association panel population.

Phenotypic evaluation of the association panel population revealed a highly significant variability for disease incidence, severity, PQ and DTF indicating the presence of considerable genetic variation among evaluated genotypes that could be exploited in breeding for resistance to ergot. The results indicated that ergot infestation was significantly influenced by year and genotype by year interaction effects, thus confirming the importance of multiple environments for germplasm evaluations at disease hotspots to identify durable and stable resistant genotypes. The observed significant mean squares for year and year by genotype interaction among genotypes for resistance to ergot traits including ergot incidence and severity as well as the different level of resistance exhibited among genotypes suggested that resistance to ergot was quantitatively inherited. In line with this, Parh et al. [30], indicated that resistance to ergot in sorghum was a polygenic trait controlled by several quantitative trait loci. McLaren and Flett [39] noted that ergot infection and pollen traits were influenced by genotype by environment interactions and these traits were controlled by quantitative trait loci.

In this study, marker trait associations analysis was performed using six ML – GWAS methods including mrMLM, FASTmrMLM, FASTmrEMMA, and pLARmEB, ISIS EM-BLASSO and pKWmEB and thirty-eight QTNs were detected by at least three methods. Of these QTNs, S4_3127636 linked with EI – 15WAP was detected by all the six methods and S9_51757008, S1_72403757 and S2_3639089 linked with ES – 15WAP, ES – 16WAP and DTF, respectively were detected simultaneously by five different methods (Table 4). The other 12 and 22 QTNs were detected by four and three different ML – GWAS methods, respectively. The results suggested that there are reliable QTNs associated with resistance to ergot in sorghum and these QTNs could be used for further studies. In this study, twenty-one QTNs being associated with ergot severity indicated that this trait was quantitatively inherited and the result of additive effect of these alleles. Similar results were observed for disease incidence as it was the result of the effects of eight QTNs (S2 Table). In line with this, Parh et al. [14] reported that resistance to ergot traits in sorghum were quantitatively inherited and controlled by several QTLs.

Of the 38 QTNs commonly identified by at least three ML – GWAS methods, 19 QTNs (10 QTNs linked with severity, 4 with incidence, 3 with DTF and 2 with PQ) had each more than 5% phenotypic effect on variability of ergot severity, incidence, DTF and PQ (Table 5) indicating QTNs with higher phenotype effects were identified in this study. QTNs, S10_36991277, S6_1811916, S1_72403757, S5_56592572, S8_40292627 exhibited the highest phenotypic expressions for ergot severity and each QTNs explained up to 12.7% of phenotypic variations in disease severity. Similarly, QTNs S1_56665164, S4_3127636, S3_64616775 and S7_10036871 associated with disease incidence explained up to 9.3% of phenotypic variations in ergot incidence (Table 5), suggesting the significance of these QTNs in resistance to ergot.

A total of 109 candidate genes linked with QTNs that have relatively higher phenotypic effects were detected. Of the 109 candidate genes, 62 genes were not characterized or unknown, while the remaining 47 genes coded for known proteins (Table 6). Functional annotation of the candidate genes showed that most of these genes encoded DNA binding proteins, NAC proteins, PMEI domain contain proteins, NB-ARC domain-containing protein and Protein kinase domain – containing proteins (Table 6). Gene ontology analysis of these proteins revealed that they were involved in different molecular and biological functions associated with resistance to a pathogen attack including DNA replication and recombination, transcription and DNA repair, magnesium ion transport, regulation of transcription and translation and transduction signaling and the transcription factors that modulate gene expressions [40]. Upon recognition of molecules released by the pathogens, plants activate a signaling cascade leading to activation of defense related genes to mount robust and quick defense responses to prevent the spread of pathogens [40,41].

Several candidate genes identified in this study including SORBI_3005G018200, SORBI_3006G012300, SORBI_3002G233800, SORBI_3002G234300, SORBI_3005G130051, SORBI_3003G254000, SORBI_3003G254100, SORBI_3003G254850, SORBI_3003G254900, SORBI_3003G255000, SORBI_3001G289900, SORBI_3004G037900, SORBI_3003G203800 and SORBI_3001G221000 (Table 6) encoded ATP, DNA and RNA binding proteins. DNA and RNA binding proteins play a key role in regulation of many cellular processes including transcription, translation, gene silencing, micro-RNA biogenesis and telomere maintenance that resulted in activation of defense responses against various pathogen invasions [42]. Additionally, these proteins were reported to play a key role in modulating gene expression, cell survival, mediating responses to various stresses and homeostasis [38]. ATP binding proteins were reported to be involved in regulating diverse biological processes such as growth, development and defense response against pathogenic infection [43].

On chromosome 5 SORBI_3005G018400 gene was collocated with the QTN S5_1649273. This gene encoded NB-ARC domain-containing proteins. The NB-ARC domain proteins are one of the three components of nucleotide-binding leucine-rich repeat (NLR) R genes which act as intracellular receptors for recognizing the invading pathogen and are responsible for defense-related signaling [44]. Previously, NB-ARC-mediated resistance has been detected against various fungal, oomycetes and bacterial pathogens [45]. Similarly, Zhang et al. [44] reported an NB-ARC domain containing disease resistance protein gene against Exserohilum turcicum in Sorghum and Maize.

SORBI_3001G323801 gene associated with QTN S4_3127636 on chromosome 4 encoded for PMEI domain-containing proteins (Table 6). These proteins were reported to be involved in various biological processes such as regulation of defense related signal pathways, increasing plant fertility and pollen viability [46]. The results of our study highlighted a negativecorrelation between pollen quantity and resistance to ergot traits (Fig 1), indicating that pollen production plays a crucial role in the dynamics of ergot infection, with resistant genotypes tend to exhibit higher pollen quantities, which could be a defense mechanism against ergot infection by ensuring rapid pollination and fertilization before the ergot infection can take hold. In line with this, Parh et al [14] reported that resistance of sorghum to ergot infection was related with the ability of plants to pollinate and fertilize rapidly before infection can occur. Prom et al [13] reported that Grif627 accession accessed from China exhibited greater ergot susceptibility because of its poor pollen viability.

SORBI_3003G254700, SORBI_3003G254300, SORBI_3003G203400, SORBI_3003G254100 and SORBI_3003G253600 genes located on chromosome 3 were predicted to code for Glycosyltransferases, Glutaredoxin dependent peroxiredoxin and Protein kinase domain – containing proteins. Glycosyltransferases and Glutaredoxin dependent peroxiredoxin proteins are major regulatory components in almost all cellular processes in eukaryotic cells regulate the activity, localization, protein–protein interactions, and other features of their target proteins [47]. It has been reported that Protein kinase domain – containing proteins contribute to the biosynthesis of plant stress or defense hormones and subsequent signaling, generation of reactive oxygen species, activation of defense-related genes, phytoalexin biosynthesis, and hypersensitive response-associated cell death [48].

Proteins containing metal ion domains such as zinc finger were reported to play crucial roles in eukaryotic cells regulating different signal transduction pathways and controlling several biological processes, such as development and programmed cell death [49]. In this study, candidate genes including SORBI_3008G109300, SORBI_3008G109400, SORBI_3003G028600, 3SORBI_3003G380100, SORBI_3002G234100, SORBI_3002G234200, SORBI_3006G012500, SORBI_3005G130100 located on chromosomes 2, 3, 5, 6 and 8 were linked with several metal ion domains containing proteins (Table 6).

NAC domain-containing proteins are transcriptional factors and function in the promoter region of different stress-related genes and promote plant defense when pathogenic infection occurs and they are central components of many aspects of the plant innate immune system, basal defense, and systemic acquired resistance [50]. In this study four candidate genes SORBI_3003G254850, SORBI_3003G254900, SORBI_3003G255000, and SORBI_3005G018500 located on chromosome 3 and 5 associated with severity were detected encoding for NAC domain-containing proteins suggesting these proteins were involved in resistance to ergot.

On chromosome three, SORBI_3003G380100 involved in pollen quantity encoded DYW deaminase domain containing proteins that belong to pentatricopeptide repeat (PRR) gene family and were reported to have RNA editing roles [44]. The expression of these proteins is directly associated with fertility restoration and increase pollen production resulting in lower ergot infections [51].

## Conclusions

Considerable genetic variation was observed among evaluated genotypes for resistance sorghum traits associated with ergot disease including incidence, severity, DTF and PQ. ML – GWAS analysis using sorghum association panel detected thirty-eight significant QTNs. Further analysis identified 19 QTNs with relatively higher phenotypic effects and 47 positional candidate genes associated with significant QTNs. The identified QTNs and candidate genes could be of immense importance for marker assisted selection in improving sorghum for resistance to ergot disease.

## Supporting information

S1 Table List of genotypes used in the study. (DOCX)
S2 Table. Common SNPs identified by at least three ML-GWAS methods
S1 Fig. QQ plots for resistance to ergot and other traits; (a) EI14WAP, (b) EI15WAP, (c) ES14WAP, (d) ES15WAP, (e) ES16WAP, (f) DTF and (g) PQ.
