# Supplementary material for: Multi-locus GWAS analysis identifies genomic regions associated with resistance to ergot (Claviceps africana) in sorghum
Source: PLoS One. 2025 Jun 23;20(6):e0325224. doi: 10.1371/journal.pone.0325224 (PMC12184897; doi:10.1371/journal.pone.0325224)
Supplement: S1 Table — (DOCX) S2 Table. Common SNPs identified by at least three ML-GWAS methods S1 Fig. QQ plots for resistance to ergot and other traits; (a) EI14WAP, (b) EI15WAP, (c) ES14WAP, (d) ES15WAP, (e) ES16WAP, (f) DTF and (g) PQ. [file pone.0325224.s001.docx]

**Supporting information**

**S1 Table. List of genotypes used in the study**

| Origin | No of genotypes | ID |
| --- | --- | --- |
| Arabia | 1 | PI533997 |
| Botswana | 1 | PI576352 |
| Brazil | 1 | PI576437 |
| Burkina Faso | 1 | PI595745 |
| Chad | 3 | PI533863, PI534037 and PI595718 |
| China | 3 | PI533980, PI576332 and PI597946 |
| Congo | 1 | PI533956 |
| Egypt | 1 | PI597945 |
| Ethiopia | 30 | PI533761, PI533792, PI533794, PI533800, PI533902, PI533919, PI533927, PI534115, PI534123, PI534124, PI534127, PI534128, PI534132, PI534133, PI534135, PI534148, PI534155, PI534157, PI576375, PI576376, PI576380, PI576381, PI576393, PI576425, PI576426, PI576428, PI595720, PI597957, PI597964 and PI597967 |
| Guatemala | 1 | PI595744 |
| India | 19 | PI533810, PI533814, PI533842, PI533855, PI533856, PI533943, PI534009, PI534021, PI534028, PI576359, PI576364, PI576366, PI576390, PI576391, PI576401, PI595699, PI597949, PI597950 |
| Japan | 4 | PI534097, PI534099, PI534101 and PI576373 |
| Kenya | 2 | PI534054 and PI534096 |
| Mali | 2 | PI597976 and PI597980 |
| Mozambique | 1 | PI533939 |
| Nigeria | 15 | PI533785, PI533807, PI533839, PI533871, PI533876, PI533877, PI534070, PI534075, PI534079, PI534088, PI534092, PI576385, PI576418, PI597960 and PI597961 |
| Pakistan | 1 | PI534114 |
| Senegal | 1 | PI595741 |
| South Africa | 8 | PI533752, PI533955, PI533961, PI533976, PI533979, PI576340, PI576345 and PI576422 |
| Sudan | 31 | PI533754, PI533758, PI533762, PI533769, PI533788, PI533789, PI533866, PI533910, PI533911, PI533912, PI533913, PI533962, PI533964, PI533985, PI533986, PI533987, PI533989, PI533991, PI533996, PI534137, PI534139, PI576387, PI576399, PI595714, PI595739, PI597952, PI597968, PI597971, PI597973 and PI597982 |
| Tanzania | 5 | PI533821, PI533822, PI533869, PI533936 and, PI533940 |
| Uganda | 14 | PI533833, PI533965, PI533967, PI533970, PI533972, PI534053, PI534104, PI534105, PI534108, PI534112, PI534117, PI534144, PI576386 and PI576435 |
| USA | 11 | PI48770, PI533948, PI533957, PI533998, PI534163 and PI552861 |
| Venezuela | 1 | PI597966 |
| West Volta | 2 | PI533766 and PI597951 |
| Zambia | 1 | PI533937 |
| Zimbabwe | 3 | PI534145, PI576339 and PI595702 |
| NA | 167 | PI152651, PI276837, PI34911, PI533755, PI533757, PI533759, PI533760, PI533776, PI533799, PI533824, PI533838, PI533841, PI533843, PI533845, PI533852, PI533878, PI533924, PI534063, PI534138, PI534167, PI542718, PI548797, PI552856, PI561073, PI561472, PI564164, PI564165, PI576130, PI585291, PI585295, PI595743, PI597965, PI597972, PI598069, PI601816, PI607931, PI609456, PI629034, PI629040, PI629059, PI641849, PI641874, PI642992, PI642998, PI65121, PI651492, PI651496, PI655970, PI655971, PI655972, PI655973,PI655974,PI655975, PI655976, PI655977, PI655978, PI655980, PI655981, PI655982, PI655983, PI655985, PI655986, PI655987, PI655988, PI655989, PI655990, PI655991, PI655992, PI655993, PI655994, PI655995, PI655997, PI655998, PI655999, PI656000, PI656001, PI656003, PI656004, PI656010, PI656011, PI656012, PI656013, PI656014, PI656015, PI656016, PI656017, PI656018, PI656019, PI656022, PI656023, PI656025, PI656026, PI656027, PI656028, PI656029, PI656030, PI656031, PI656032, PI656033, PI656034, PI656035, PI656036, PI656037, PI656038, PI656041, PI656043, PI656044, I656046, PI656047, PI656048, PI656049, PI656050, PI656051, PI656052, PI656053, PI656055, PI656056, PI656058, PI656059, PI656062, PI656064, PI656065, PI656066, PI656067, PI656068, PI656070, PI656071, PI656072, PI656074, PI656075, PI656076, PI656077, PI656078, PI656079, PI656080, PI656081, PI656082, PI656083, PI656086, PI656087, PI656088, PI656089, PI656090, PI656091, PI656092, PI656093, PI656094, PI656095, PI656096, PI656097, PI656101, PI656102, PI656104, PI656105, PI656106, PI656107, PI656108, PI656109, PI656110, PI656111, PI656112, PI656114, PI656116, PI656118, PI656119, PI656120 and PI656121 |

**S2 Table. Common SNPs identified by at least three ML - GWAS methods**

| Trait | Method | QTNs | Chr | Position (bp) | LOD score | r^2^ (%) | MAF | Alleles |
| --- | --- | --- | --- | --- | --- | --- | --- | --- |
| ES –14WAP | 1, 4, 5, 6 | S10_36991277 | 10 | 36991277 | 3.27 - 7.44 | 2.15 - 12.7 | 0.21 | G/A |
|  | 1, 2, 4, 6 | S4_9623316 | 4 | 9623316 | 3.51 - 4.76 | 1.84 - 3.54 | 0.11 | C/A |
|  | 1, 2, 4 | S5_1649273 | 5 | 1649273 | 3.34 - 7.37 | 4.85 - 7.90 | 0.30 | T/C |
|  | 2, 4, 6 | S8_49792801 | 8 | 49792801 | 4.26 - 6.13 | 2.35 - 3.27 | 0.17 | T/C |
|  | 2, 5, 6 | S10_52675648 | 10 | 52675648 | 3.54 - 6.43 | 1.60 - 2.64 | 0.17 | C/T |
| ES – 15WAP | 1 – 5 | S9_51757008 | 9 | 51757008 | 3.32 - 6.54 | 2.79 - 4.52 | 0.38 | A/C |
|  | 1, 2, 4, 5 | S3_70612486 | 3 | 70612486 | 3.32 - 5.97 | 1.70 - 3.27 | 0.40 | T/G |
|  | 1, 2, 4, 5 | S1_66757591 | 1 | 66757591 | 4.65 - 9.12 | 1.55 - 3.35 | 0.50 | A/A |
|  | 1, 2, 4, 5 | S6_1811916 | 6 | 1811916 | 6.55 - 19.21 | 4.54 - 11.6 | 0.47 | T/C |
|  | 1, 2, 4, 5 | S8_40292627 | 8 | 40292627 | 5.46 - 14.98 | 5.49 - 10.8 | 0.23 | T/C |
|  | 2, 4, 6 | S9_56646260 | 9 | 56646260 | 5.08 - 5.48 | 1.76 - 3.54 | 0.13 | G/A |
|  | 1, 2, 5 | S5_48357193 | 5 | 48357193 | 3.46 - 7.85 | 2.54 - 3.96 | 0.10 | A/C |
|  | 4, 5, 6 | S1_1554888 | 1 | 1554888 | 3.32 - 5.52 | 0.83 - 4.28 | 0.41 | C/A |
|  | 1, 5, 6 | S1_26468239 | 1 | 26468239 | 4.32 - 7.45 | 2.29 - 5.13 | 0.10 | G/A |
|  | 1, 2, 6 | S1_57509661 | 1 | 57509661 | 3.52 - 5.30 | 3.42 - 4.69 | 0.17 | G/A |
|  | 2, 4, 5 | S2_62482269 | 2 | 62482269 | 3.71 - 6.59 | 3.44 - 7.04 | 0.17 | T/C |
| ES – 16WAP | 1, 2, 4, 5, 6 | S1_72403757 | 1 | 72403757 | 4.85 - 10.14 | 3.76 - 11.4 | 0.21 | T/A |
|  | 1, 3, 4, 6 | S5_1649273 | 5 | 1649273 | 3.11 - 4.99 | 3.17 - 5.95 | 0.30 | T/C |
|  | 2, 4, 5, 6 | S6_41603668 | 6 | 41603668 | 4.61 - 10.7 | 4.90 - 7.44 | 0.07 | A/G |
|  | 1, 2, 4 | S5_56592572 | 5 | 56592572 | 3.44 - 6.46 | 1.84 - 10.2 | 0.17 | C/T |
|  | 3, 4, 6 | S5_2816369 | 5 | 2816369 | 3.08 - 4.27 | 1.44 - 4.28 | 0.44 | C/G |
|  | 1, 2, 6 | S3_59282235 | 3 | 59282235 | 3.02 - 8.71 | 2.14 - 6.66 | 0.14 | G/T |
| EI – 14WAP | 4, 5, 6 | S1_56665164 | 1 | 56665164 | 3.57 - 9.12 | 2.16 - 9.32 | 0.46 | G/C |
|  | 1, 2, 5 | S2_76890918 | 2 | 76890918 | 3.89 - 5.05 | 2.38 - 4.57 | 0.42 | T/C |
| EI – 15WAP | 1 – 6 | S4_3127636 | 4 | 3127636 | 4.45 - 7.05 | 2.37 - 5.63 | 0.44 | C/T |
|  | 2, 3, 4, 6 | S1_61119088 | 1 | 61119088 | 4.91 - 7.20 | 2.63 - 3.34 | 0.25 | C/T |
|  | 2, 3, 5 | S3_64616775 | 3 | 64616775 | 3.81 - 6.24 | 2.40 - 4.97 | 0.44 | C/T |
|  | 1, 2, 6 | S6_28535393 | 6 | 28535393 | 3.86 - 5.23 | 2.70 - 3.80 | 0.26 | T/C |
|  | 1, 2, 3 | S7_10036871 | 7 | 10036871 | 3.38 - 3.65 | 1.74 - 4.88 | 0.17 | A/G |
|  | 1, 2, 6 | S8_1255457 | 8 | 1255457 | 3.10 - 3.84 | 1.91 - 3.19 | 0.49 | T/G |
| DTF | 1, 2, 3, 4, 6 | S2_3639089 | 2 | 3639089 | 4.41 - 7.66 | 1.49 - 4.82 | 0.10 | C/T |
|  | 1, 2, 4, 6 | S1_20799733 | 1 | 20799733 | 5.83 - 8.28 | 5.95 - 11.3 | 0.20 | C/A |
|  | 1, 2, 3, 5 | S3_2543818 | 3 | 2543818 | 3.54 - 6.74 | 2.85 - 5.97 | 0.21 | A/G |
|  | 1, 3, 4 | S10_53061826 | 10 | 53061826 | 3.29 - 8.15 | 1.59 - 4.77 | 0.24 | C/A |
|  | 2, 4, 6 | S4_67386825 | 4 | 67386825 | 3.46 - 5.48 | 1.98 - 3.43 | 0.32 | A/G |
|  | 1, 3, 5 | S8_50806664 | 8 | 50806664 | 4.68 - 9.05 | 3.33 - 6.29 | 0.41 | C/A |
| PQ | 2, 4, 5, 6 | S9_8797645 | 9 | 8797645 | 3.12 - 5.57 | 0.83 - 2.18 | 0.17 | C/T |
|  | 1, 2, 4 | S3_53363038 | 3 | 53363038 | 3.44 - 4.58 | 2.30 - 6.52 | 0.13 | A/G |
|  | 4, 5, 6 | S3_69400934 | 3 | 69400934 | 3.84 - 8.18 | 2.91 - 7.66 | 0.17 | G/T |

1, mrMLM; 2, FASTmrMLM; 3, FASTmrEMMA; 4, pLARmEB; 5, pKWmEB; 6, ISIS EM-BLASSO, EI – ergot incidence, ES – ergot severity, WAP – weeks after planting, DTF – days to 50% flowering, PQ – pollen quantity, Chr – chromosome, QTNs – quantitative trait nucleotides, MAF – minor allele frequency and LOD – logarithms of odds


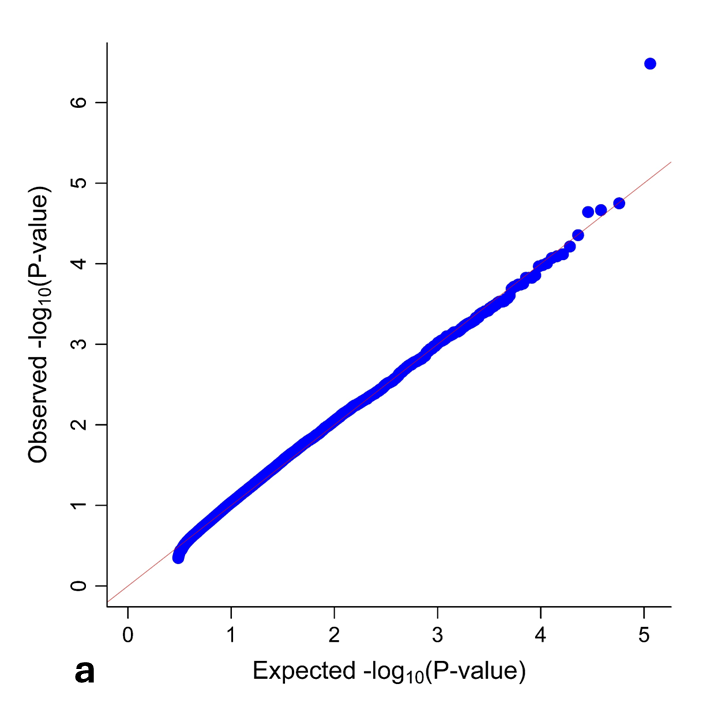

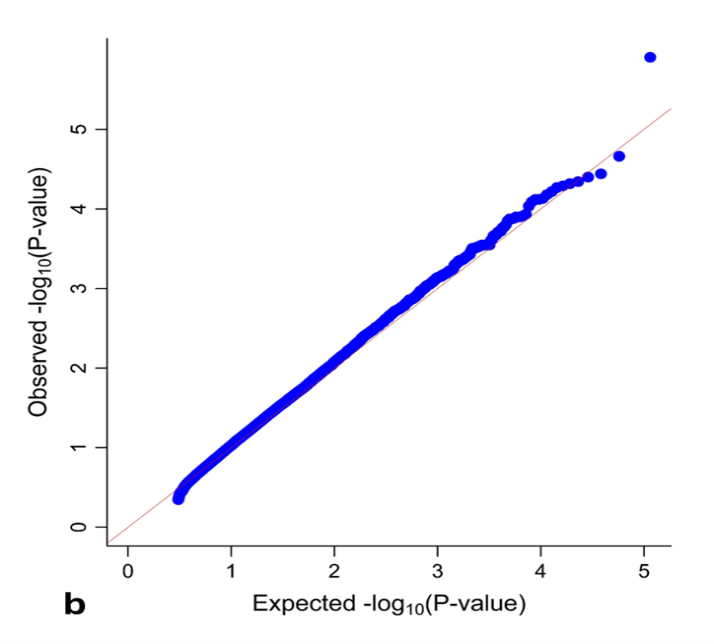


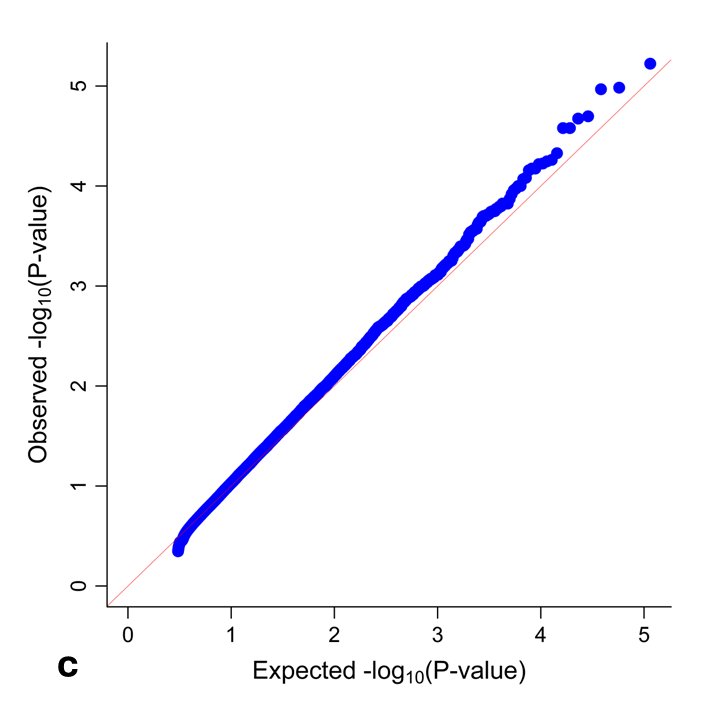

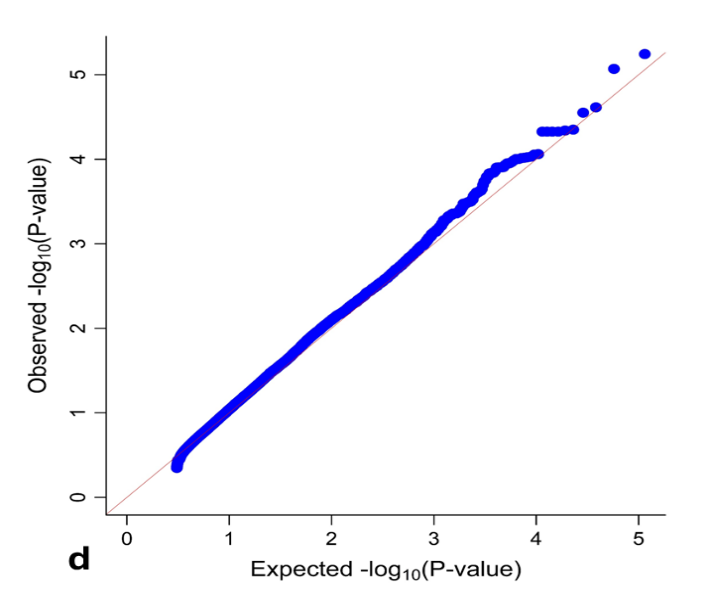


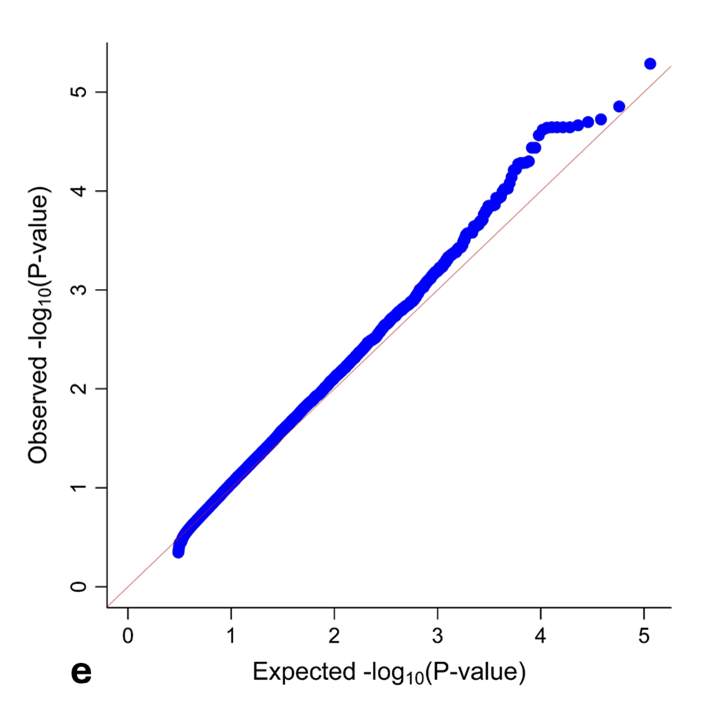

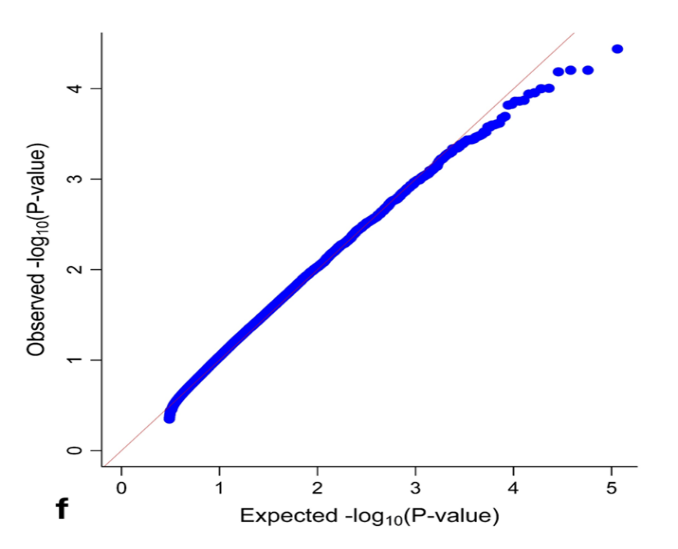


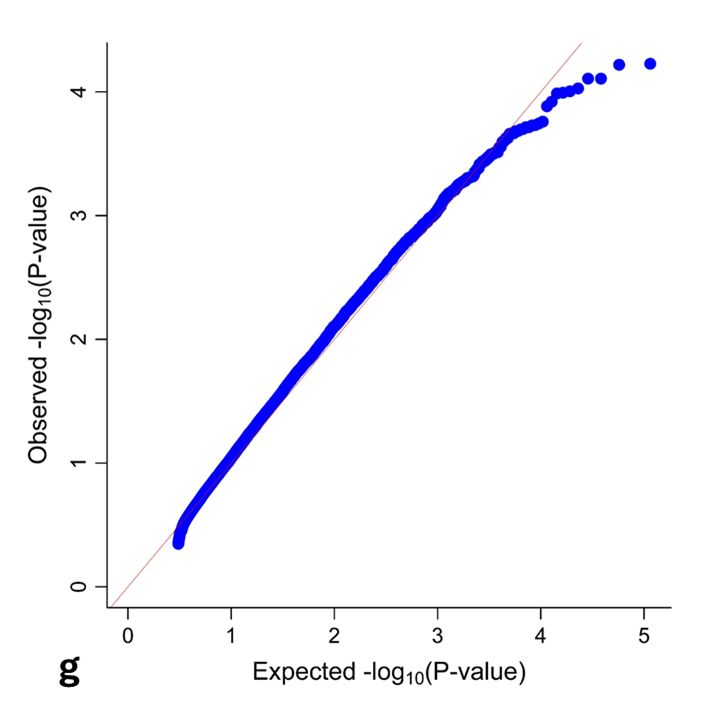


**S1 Fig. QQ plots for resistance to ergot and other traits; (a) EI14WAP, (b) EI15WAP, (c) ES14WAP, (d) ES15WAP, (e) ES16WAP, (f) DTF and (g) PQ.**

**S3 Table. Mean value of the response of SAP population to ergot infection**

| Genotype | DTF | PQ | ES - 14WAP | ES - 15WAP | ES - 16WAP | EI - 14WAP | EI - 15WAP | EI - 16WAP |
| --- | --- | --- | --- | --- | --- | --- | --- | --- |
| SAP_PI152595 | 79.27 | 6.75 | 2.07 | 2.58 | 2.55 | 27.76 | 42.93 | 46.94 |
| SAP_PI152651 | 78.51 | 6.43 | 2.51 | 3.12 | 3.24 | 53.73 | 76.61 | 88.35 |
| SAP_PI276837 | 94.26 | 5.77 | 1.51 | 2.31 | 2.83 | NA | 42.35 | 60.27 |
| SAP_PI34911 | 69.93 | 6.76 | 2.21 | 2.91 | 2.83 | 29.51 | 55.16 | 50.01 |
| SAP_PI48770 | 80.36 | 6.72 | 2.20 | 2.40 | 2.34 | 31.46 | 44.28 | 50.11 |
| SAP_PI533750 | 80.15 | 7.19 | 2.67 | 3.05 | 3.10 | 37.77 | 64.48 | 73.49 |
| SAP_PI533752 | 80.64 | 7.31 | 2.64 | 2.81 | 2.86 | 29.79 | 44.56 | 48.49 |
| SAP_PI533754 | 84.29 | 6.49 | 3.79 | 3.89 | 4.04 | 86.94 | 99.81 | 103.77 |
| SAP_PI533755 | 81.17 | 6.69 | 2.85 | 3.02 | 2.98 | 76.24 | 93.24 | 92.98 |
| SAP_PI533757 | 73.30 | 6.85 | 2.86 | 3.55 | 3.52 | 55.78 | 74.57 | 76.42 |
| SAP_PI533758 | 72.15 | 5.88 | 2.49 | 2.80 | 2.87 | 27.12 | 43.28 | 58.19 |
| SAP_PI533759 | 86.76 | 6.28 | 2.89 | 3.06 | 3.17 | 39.92 | 66.24 | 68.39 |
| SAP_PI533760 | 95.82 | 5.95 | NA | NA | NA | NA | NA | NA |
| SAP_PI533761 | 87.32 | 6.73 | 2.17 | 2.75 | 2.88 | 37.24 | 49.77 | 74.85 |
| SAP_PI533766 | 74.67 | 6.80 | 2.79 | 3.26 | 3.42 | 49.84 | 81.17 | 88.09 |
| SAP_PI533769 | 76.05 | 6.60 | 2.30 | 2.69 | 2.70 | 39.22 | 72.38 | 76.25 |
| SAP_PI533776 | 80.58 | 6.83 | 2.91 | 3.23 | 3.24 | 63.40 | 86.45 | 85.92 |
| SAP_PI533785 | 85.04 | 6.67 | 2.94 | 4.34 | 4.45 | 54.03 | 82.42 | 84.08 |
| SAP_PI533788 | 77.06 | 6.84 | 2.68 | 3.48 | 3.41 | 48.44 | 58.55 | 62.52 |
| SAP_PI533789 | 76.90 | 6.55 | 2.30 | 2.68 | 2.75 | 31.06 | 59.43 | 66.80 |
| SAP_PI533792 | 74.43 | 7.15 | 2.77 | 3.23 | 3.29 | 38.84 | 61.46 | 69.76 |
| SAP_PI533794 | 83.59 | 7.00 | 2.22 | 2.55 | 2.63 | 29.39 | 43.53 | 52.66 |
| SAP_PI533799 | 84.31 | 6.36 | 2.28 | 2.52 | 2.59 | 65.35 | 86.99 | 93.48 |
| SAP_PI533800 | 82.94 | 6.68 | 2.32 | 2.94 | 2.92 | 32.17 | 56.54 | 59.73 |
| SAP_PI533807 | 66.88 | 7.19 | 2.53 | 2.72 | 2.66 | 42.18 | 62.40 | 78.31 |
| SAP_PI533810 | 89.95 | 6.01 | 2.61 | 3.20 | 3.99 | 65.88 | 101.92 | 101.40 |
| SAP_PI533814 | 88.54 | 6.76 | 2.32 | 3.00 | 3.07 | 34.76 | 57.09 | 61.52 |
| SAP_PI533821 | 76.76 | 6.24 | 2.42 | 3.12 | 3.18 | 41.19 | 74.01 | 78.63 |
| SAP_PI533822 | 83.54 | 6.97 | 2.36 | 2.97 | 3.11 | 40.92 | 82.83 | 88.68 |
| SAP_PI533824 | 84.32 | 6.84 | 2.35 | 2.81 | 3.27 | 26.07 | 48.87 | 57.79 |
| SAP_PI533833 | 76.00 | 6.81 | 4.31 | 4.44 | 4.46 | 87.71 | 89.05 | 92.99 |
| SAP_PI533838 | 74.54 | 6.30 | 3.05 | 3.70 | 4.10 | 65.60 | 90.38 | 95.83 |
| SAP_PI533839 | 92.56 | 6.82 | 2.47 | 3.03 | 3.06 | 34.88 | 53.18 | 60.83 |
| SAP_PI533841 | 79.23 | 6.58 | 3.04 | 3.63 | 3.89 | 63.43 | 84.49 | 89.68 |
| SAP_PI533842 | 97.09 | 7.10 | 2.63 | 2.94 | 3.09 | 40.32 | 59.43 | 72.36 |
| SAP_PI533843 | 83.24 | 6.93 | 3.29 | 4.01 | 4.18 | 75.48 | 90.22 | 97.04 |
| SAP_PI533845 | 84.57 | 6.67 | 2.41 | 2.66 | 2.71 | 34.18 | 56.60 | 66.21 |
| SAP_PI533852 | 86.10 | 6.74 | 2.71 | 3.41 | 3.40 | 48.92 | 64.82 | 69.90 |
| SAP_PI533855 | 89.75 | 6.08 | 3.16 | 4.02 | 4.29 | 75.83 | 91.89 | 101.34 |
| SAP_PI533856 | 92.24 | 6.79 | 2.16 | 2.83 | 2.82 | 43.08 | 60.61 | 68.56 |
| SAP_PI533863 | 78.30 | 6.51 | 2.80 | 3.69 | 3.71 | 62.55 | 88.56 | 90.19 |
| SAP_PI533866 | 80.66 | 6.99 | 2.58 | 3.03 | 3.29 | 67.22 | 87.54 | 83.35 |
| SAP_PI533869 | 82.15 | 6.26 | 2.35 | 3.10 | 3.49 | 31.54 | 45.55 | 65.06 |
| SAP_PI533871 | 78.46 | 6.65 | 2.08 | 2.74 | 2.72 | 24.34 | 40.70 | 46.01 |
| SAP_PI533876 | 76.51 | 7.52 | 2.11 | 2.50 | 2.53 | 15.97 | 29.69 | 34.98 |
| SAP_PI533877 | 76.00 | 6.50 | 2.98 | 3.00 | 3.09 | 62.05 | 61.23 | 66.88 |
| SAP_PI533878 | 87.88 | 6.27 | 2.63 | 3.78 | 3.92 | 59.19 | 101.24 | 101.13 |
| SAP_PI533902 | 84.10 | 7.30 | 2.79 | 3.67 | 3.70 | 42.21 | 81.85 | 83.10 |
| SAP_PI533910 | 82.39 | 6.64 | 2.05 | 2.51 | 2.52 | 29.36 | 60.91 | 71.60 |
| SAP_PI533911 | 78.79 | 6.63 | 2.14 | 2.54 | 2.53 | 39.23 | 50.62 | 50.89 |
| SAP_PI533912 | 65.46 | 6.59 | 2.51 | 2.73 | 2.92 | 52.58 | 61.85 | 72.32 |
| SAP_PI533913 | 79.05 | 6.61 | 2.00 | 2.47 | 2.59 | 50.90 | 64.30 | 70.65 |
| SAP_PI533919 | 81.34 | 6.66 | 2.84 | 3.36 | 3.60 | 68.98 | 83.23 | 88.60 |
| SAP_PI533924 | 87.68 | 6.98 | 3.10 | 3.55 | 3.52 | 49.35 | 74.84 | 78.05 |
| SAP_PI533927 | 89.42 | 6.62 | 2.20 | 2.92 | 3.10 | 23.65 | 44.95 | 59.53 |
| SAP_PI533936 | 82.69 | 6.63 | 2.51 | 2.48 | 2.45 | 29.58 | 34.60 | 42.67 |
| SAP_PI533937 | 79.59 | 6.64 | 2.39 | 3.12 | 2.90 | 31.78 | 60.91 | 74.61 |
| SAP_PI533939 | 80.41 | 6.29 | 2.66 | 3.17 | 3.36 | 43.68 | 59.01 | 66.81 |
| SAP_PI533940 | 81.43 | 6.74 | 3.34 | 4.05 | 4.19 | 47.61 | 80.93 | 98.65 |
| SAP_PI533943 | 85.25 | 6.81 | 2.61 | 3.19 | 3.20 | 58.26 | 75.06 | 77.88 |
| SAP_PI533948 | 89.34 | 6.57 | 2.17 | 2.65 | 2.66 | 46.35 | 71.98 | 78.62 |
| SAP_PI533955 | 81.03 | 6.69 | 2.06 | 2.83 | 2.88 | 52.19 | 60.79 | 73.75 |
| SAP_PI533956 | 79.14 | 6.65 | 2.54 | 3.09 | 3.19 | 30.39 | 46.31 | 60.21 |
| SAP_PI533957 | 71.12 | 7.13 | 3.40 | 3.73 | 3.74 | 44.71 | 60.59 | 80.69 |
| SAP_PI533961 | 70.63 | 7.00 | 1.98 | 2.57 | 2.60 | 21.90 | 43.86 | 49.76 |
| SAP_PI533962 | 73.39 | 7.46 | 2.09 | 2.35 | 2.30 | 10.58 | 27.50 | 32.49 |
| SAP_PI533964 | 79.71 | 6.76 | 2.14 | 2.30 | 2.32 | 23.19 | 33.25 | 35.44 |
| SAP_PI533965 | 79.21 | 7.43 | 2.17 | 2.34 | 2.36 | 21.38 | 33.10 | 42.07 |
| SAP_PI533967 | 76.51 | 7.27 | 2.52 | 2.90 | 2.83 | 45.25 | 54.48 | 59.36 |
| SAP_PI533970 | 80.50 | 6.72 | 2.32 | 2.81 | 2.92 | 46.04 | 71.58 | 77.45 |
| SAP_PI533972 | 82.87 | 7.03 | 2.11 | 2.50 | 2.51 | 30.91 | 42.36 | 46.09 |
| SAP_PI533976 | 73.84 | 7.48 | 2.63 | 3.28 | 3.81 | 43.07 | 60.96 | 71.91 |
| SAP_PI533979 | 94.84 | 6.75 | 1.98 | 2.71 | 2.81 | 24.64 | 66.64 | 72.50 |
| SAP_PI533980 | 78.04 | 6.32 | 2.35 | 3.07 | 2.70 | 62.48 | 73.93 | 75.57 |
| SAP_PI533985 | 80.09 | 6.34 | 1.84 | 1.89 | 1.92 | 12.60 | 36.06 | 39.91 |
| SAP_PI533986 | 81.98 | 6.27 | 2.53 | 3.12 | 2.96 | 37.63 | 72.90 | 81.20 |
| SAP_PI533987 | 76.79 | 6.56 | 2.42 | 2.94 | 2.93 | 55.54 | 72.97 | 73.06 |
| SAP_PI533989 | 73.58 | 7.51 | 2.57 | 2.77 | 2.99 | 40.51 | 63.79 | 75.50 |
| SAP_PI533991 | 73.80 | 6.90 | 2.01 | 2.68 | 2.70 | 20.63 | 35.04 | 43.07 |
| SAP_PI533996 | 77.60 | 6.74 | 2.81 | 3.16 | 3.16 | 42.58 | 68.90 | 72.97 |
| SAP_PI533997 | 85.77 | 6.66 | 3.48 | 3.86 | 4.04 | 77.84 | 101.48 | 101.08 |
| SAP_PI533998 | 82.91 | 6.80 | 2.79 | 3.22 | 3.36 | 48.97 | 75.76 | 84.94 |
| SAP_PI534009 | 78.91 | 6.75 | 3.78 | 4.72 | 4.25 | 80.40 | 106.05 | 105.28 |
| SAP_PI534021 | NA | NA | NA | 1.74 | 1.74 | NA | 47.07 | 45.23 |
| SAP_PI534028 | 89.48 | 6.67 | 2.35 | 2.51 | 2.76 | 39.14 | 64.22 | 73.23 |
| SAP_PI534037 | NA | 6.34 | 2.57 | 3.08 | 3.71 | 19.70 | 48.94 | 76.36 |
| SAP_PI534053 | 80.30 | 6.49 | 2.50 | 3.14 | 3.16 | 45.25 | 59.46 | 69.58 |
| SAP_PI534054 | 80.01 | 6.50 | 2.00 | 2.51 | 2.44 | 30.97 | 44.55 | 51.34 |
| SAP_PI534063 | 74.01 | 6.72 | 1.64 | 2.22 | 2.60 | 6.29 | 22.68 | 42.50 |
| SAP_PI534070 | 79.75 | 6.58 | 1.99 | 2.33 | 2.24 | 26.32 | 34.83 | 43.36 |
| SAP_PI534075 | 70.87 | 6.83 | NA | NA | 3.45 | NA | NA | 65.65 |
| SAP_PI534079 | 70.70 | 6.85 | 2.27 | 2.55 | 2.58 | 23.66 | 34.61 | 47.30 |
| SAP_PI534088 | 70.87 | 6.62 | 1.43 | 2.20 | 3.15 | NA | 45.78 | 93.09 |
| SAP_PI534092 | 100.21 | 6.03 | 2.41 | 2.73 | 3.00 | 83.58 | 100.67 | 99.80 |
| SAP_PI534096 | 77.65 | 6.31 | 1.95 | 2.36 | 2.45 | 24.19 | 39.89 | 47.18 |
| SAP_PI534097 | 83.91 | 7.28 | 2.44 | 2.39 | 2.51 | 45.54 | 61.15 | 54.33 |
| SAP_PI534099 | 68.99 | 7.09 | 2.95 | 3.34 | 3.42 | 53.18 | 64.95 | 66.26 |
| SAP_PI534101 | 75.51 | 7.07 | 2.47 | 2.83 | 2.85 | 30.26 | 49.17 | 57.62 |
| SAP_PI534104 | 78.86 | 6.68 | 2.49 | 3.03 | 3.10 | 39.58 | 53.33 | 69.23 |
| SAP_PI534105 | 72.12 | 7.92 | 2.14 | 2.57 | 2.58 | 30.32 | 52.51 | 59.00 |
| SAP_PI534108 | 81.45 | 6.84 | 2.34 | 2.31 | 2.43 | 36.32 | 56.78 | 82.74 |
| SAP_PI534112 | 77.40 | 6.95 | 2.04 | 2.68 | 2.61 | 21.13 | 41.99 | 45.98 |
| SAP_PI534114 | 76.47 | 6.84 | 2.04 | 2.72 | 2.78 | 27.82 | 37.64 | 47.14 |
| SAP_PI534115 | 86.09 | 7.11 | 2.15 | 2.63 | 2.50 | 17.22 | 32.28 | 29.72 |
| SAP_PI534117 | 78.32 | 6.78 | 3.17 | 3.79 | 3.95 | 69.78 | 73.51 | 83.91 |
| SAP_PI534123 | 74.67 | 7.30 | 1.94 | 2.34 | 2.35 | 34.67 | 47.52 | 51.48 |
| SAP_PI534124 | 83.04 | 6.74 | 2.36 | 3.93 | 4.19 | 35.78 | 58.33 | 67.82 |
| SAP_PI534127 | 75.09 | 6.88 | 3.43 | 3.76 | 3.84 | 53.48 | 79.54 | 85.75 |
| SAP_PI534128 | 83.27 | 6.84 | 2.42 | 2.91 | 3.04 | 42.43 | 60.42 | 76.01 |
| SAP_PI534132 | 71.47 | 6.59 | 2.71 | 4.08 | 4.12 | 35.28 | 86.87 | 86.02 |
| SAP_PI534133 | 87.97 | 6.61 | 2.42 | 2.74 | 2.68 | 33.54 | 46.49 | 68.40 |
| SAP_PI534135 | 90.61 | 7.01 | 2.04 | 2.97 | 3.16 | 55.92 | 72.26 | 80.26 |
| SAP_PI534137 | NA | 6.88 | NA | NA | 3.56 | NA | NA | 75.40 |
| SAP_PI534138 | 78.02 | 5.76 | 2.98 | 3.49 | 3.48 | 67.33 | 79.36 | 80.36 |
| SAP_PI534139 | 72.66 | 5.90 | 3.10 | 3.54 | 3.55 | 57.52 | 72.63 | 74.37 |
| SAP_PI534144 | 76.13 | 6.59 | 3.70 | 4.05 | 4.13 | 77.60 | 89.71 | 98.52 |
| SAP_PI534145 | 75.47 | 6.49 | 2.11 | 2.30 | 2.32 | 17.23 | 24.92 | 31.34 |
| SAP_PI534148 | 79.43 | 6.36 | 1.99 | 2.31 | 2.30 | 25.56 | 39.66 | 42.64 |
| SAP_PI534155 | 80.65 | 6.81 | 2.74 | 3.31 | 3.36 | 45.82 | 66.53 | 73.96 |
| SAP_PI534157 | 89.98 | 7.02 | 2.93 | 3.18 | 3.19 | 76.73 | 77.17 | 90.91 |
| SAP_PI534163 | 77.89 | 6.79 | 1.95 | 2.48 | 2.44 | 24.07 | 42.02 | 50.25 |
| SAP_PI534167 | 84.95 | 7.35 | 2.22 | 2.51 | 2.51 | 28.64 | 36.30 | 39.97 |
| SAP_PI542718 | 84.14 | 6.27 | 3.65 | 4.64 | 4.64 | 74.44 | 102.17 | 101.52 |
| SAP_PI548797 | 80.46 | 6.85 | 2.20 | 2.19 | 2.19 | 31.68 | 37.37 | 47.04 |
| SAP_PI552856 | 75.45 | 6.75 | NA | NA | NA | NA | NA | NA |
| SAP_PI552861 | 84.01 | 6.79 | 2.39 | 2.10 | 2.45 | 70.19 | 68.82 | 94.24 |
| SAP_PI561073 | NA | 6.10 | NA | 2.45 | 2.56 | NA | 56.40 | 72.50 |
| SAP_PI561472 | 83.47 | 6.87 | 2.22 | 2.28 | 2.57 | 47.98 | 55.81 | 84.39 |
| SAP_PI564164 | 93.71 | 6.82 | 2.24 | 2.67 | 2.75 | 65.39 | 81.54 | 95.20 |
| SAP_PI564165 | 85.95 | 6.47 | 2.29 | 3.04 | 3.00 | 33.93 | 70.07 | 89.13 |
| SAP_PI576130 | 86.79 | 6.28 | 1.99 | 2.46 | 2.42 | 24.03 | 47.40 | 51.90 |
| SAP_PI576332 | 77.65 | 6.58 | 2.70 | 3.04 | 2.95 | 36.24 | 54.84 | 64.39 |
| SAP_PI576333 | 83.40 | 6.66 | 2.22 | 2.50 | 2.55 | 33.26 | 47.79 | 62.32 |
| SAP_PI576339 | 85.07 | 6.51 | 2.06 | 2.59 | 2.48 | 27.03 | 55.46 | 62.76 |
| SAP_PI576340 | 79.09 | 6.49 | 2.34 | 2.72 | 3.07 | 49.54 | 40.24 | 76.93 |
| SAP_PI576345 | 86.21 | 6.88 | 1.99 | 2.35 | 2.37 | 14.26 | 35.79 | 39.15 |
| SAP_PI576347 | 79.68 | 7.36 | 2.02 | 2.38 | 2.56 | 27.80 | 44.09 | 53.72 |
| SAP_PI576348 | 86.65 | 6.66 | 2.00 | 2.46 | 2.74 | 69.80 | 97.75 | 99.45 |
| SAP_PI576349 | 83.00 | 6.90 | 2.37 | 2.39 | 2.21 | 38.10 | 59.22 | 56.17 |
| SAP_PI576352 | 99.00 | 6.83 | 2.85 | 3.19 | 3.19 | 66.34 | 102.39 | 100.89 |
| SAP_PI576359 | NA | 6.21 | NA | NA | 3.20 | NA | NA | 56.56 |
| SAP_PI576364 | 79.63 | 7.02 | 2.38 | 2.81 | 2.79 | 37.76 | 60.60 | 71.21 |
| SAP_PI576366 | 80.83 | 6.49 | 4.24 | 4.89 | 4.75 | 86.14 | 103.21 | 102.37 |
| SAP_PI576373 | 84.09 | 6.64 | 3.94 | 4.33 | 4.40 | 83.57 | 88.52 | 93.46 |
| SAP_PI576375 | 74.53 | 6.77 | 2.60 | 2.52 | 2.60 | 30.67 | 46.95 | 51.94 |
| SAP_PI576376 | 85.72 | 6.98 | 2.65 | 3.18 | 3.24 | 50.25 | 76.14 | 82.58 |
| SAP_PI576380 | 76.98 | 6.63 | 1.88 | 2.61 | 2.44 | 23.23 | 59.61 | 58.74 |
| SAP_PI576381 | 73.27 | 6.58 | 2.65 | 3.08 | 3.76 | 64.01 | 62.05 | 78.33 |
| SAP_PI576385 | 76.39 | 6.45 | 2.91 | 3.09 | 3.65 | 72.77 | 90.22 | 97.15 |
| SAP_PI576386 | 78.56 | 6.39 | 2.50 | 3.30 | 3.35 | 52.54 | 82.06 | 88.49 |
| SAP_PI576387 | 91.50 | 6.81 | 2.86 | 3.32 | 3.54 | 57.62 | 98.85 | 97.66 |
| SAP_PI576390 | 75.32 | 6.60 | 2.38 | 2.86 | 2.90 | 60.33 | 69.61 | 76.35 |
| SAP_PI576391 | 88.13 | 7.17 | 3.51 | 3.82 | 4.06 | 74.85 | 95.16 | 95.17 |
| SAP_PI576393 | 85.61 | 6.56 | 3.02 | 4.03 | 4.04 | 38.25 | 75.66 | 81.71 |
| SAP_PI576399 | 75.11 | 6.95 | 2.47 | 3.06 | 3.13 | 41.00 | 71.96 | 75.63 |
| SAP_PI576401 | 91.37 | 6.44 | 2.29 | 3.00 | 3.56 | 71.52 | 67.21 | 87.09 |
| SAP_PI576418 | 85.86 | 7.03 | 1.95 | 2.22 | 2.22 | 43.37 | 60.37 | 63.71 |
| SAP_PI576422 | 91.99 | 6.63 | 2.10 | 2.38 | 2.36 | 42.86 | 53.86 | 56.83 |
| SAP_PI576425 | 83.09 | 6.99 | 2.60 | 3.17 | 3.42 | 57.14 | 80.98 | 96.42 |
| SAP_PI576426 | 90.73 | 7.04 | 2.14 | 2.63 | 2.64 | 65.26 | 62.62 | 70.83 |
| SAP_PI576428 | 90.67 | 6.99 | 2.02 | 2.33 | 2.44 | 47.13 | 59.37 | 66.32 |
| SAP_PI576435 | 75.06 | 7.26 | 2.06 | 2.09 | 2.08 | 25.25 | 36.05 | 48.63 |
| SAP_PI576437 | 79.28 | 6.55 | 2.22 | 2.49 | 2.51 | 30.33 | 43.90 | 51.62 |
| SAP_PI585291 | 77.45 | 6.54 | 2.14 | 2.60 | 2.37 | 23.43 | 42.67 | 49.83 |
| SAP_PI585295 | 84.80 | 6.76 | 2.41 | 2.36 | 2.69 | 79.87 | 86.18 | 102.64 |
| SAP_PI595699 | 85.33 | 6.49 | 2.51 | 2.90 | 3.04 | 52.88 | 69.72 | 85.91 |
| SAP_PI595702 | 78.92 | 7.21 | 2.11 | 2.63 | 2.60 | 21.76 | 48.78 | 53.58 |
| SAP_PI595714 | 85.02 | 7.10 | 2.40 | 2.84 | 2.94 | 30.35 | 50.73 | 53.80 |
| SAP_PI595718 | 74.74 | 6.83 | 2.13 | 2.49 | 2.51 | 51.23 | 57.50 | 60.69 |
| SAP_PI595720 | 74.41 | 6.92 | 2.38 | 2.96 | 2.95 | 42.00 | 66.29 | 70.79 |
| SAP_PI595739 | 79.20 | 6.70 | 2.36 | 2.97 | 3.12 | 53.02 | 60.71 | 85.07 |
| SAP_PI595741 | 62.90 | 6.27 | NA | 1.87 | 1.95 | NA | 37.57 | 53.14 |
| SAP_PI595743 | 73.29 | 6.59 | 2.14 | 2.55 | 2.45 | 25.16 | 41.00 | 45.28 |
| SAP_PI595744 | 86.82 | 6.45 | 2.90 | 3.24 | 3.41 | 66.44 | 80.21 | 85.77 |
| SAP_PI595745 | 82.07 | 6.77 | 1.87 | 2.03 | 2.10 | 45.27 | 35.24 | 53.79 |
| SAP_PI597945 | 82.76 | 6.69 | 2.32 | 2.68 | 2.73 | 38.63 | 57.93 | 71.28 |
| SAP_PI597946 | 79.08 | 6.72 | 2.30 | 2.61 | 3.18 | 25.12 | 52.83 | 63.52 |
| SAP_PI597949 | 84.48 | 7.22 | 3.02 | 3.61 | 3.71 | 48.49 | 81.20 | 89.83 |
| SAP_PI597950 | 79.00 | 6.87 | 2.65 | 3.13 | 2.94 | 49.45 | 61.21 | 71.34 |
| SAP_PI597951 | 72.67 | 6.03 | 2.98 | 3.71 | 3.72 | 64.52 | 92.11 | 90.47 |
| SAP_PI597952 | 74.83 | 6.78 | 3.48 | 3.68 | 3.67 | 70.60 | 79.29 | 97.22 |
| SAP_PI597957 | 82.25 | 6.70 | 2.47 | 2.75 | 3.24 | 34.27 | 65.17 | 87.81 |
| SAP_PI597960 | 91.67 | 6.56 | 2.34 | 2.86 | 2.61 | 63.29 | 59.70 | 71.63 |
| SAP_PI597961 | 77.20 | 7.29 | 2.54 | 3.23 | 3.35 | 36.95 | 76.02 | 74.13 |
| SAP_PI597964 | 82.52 | 6.60 | 2.58 | 2.61 | 2.62 | 38.29 | 47.10 | 57.04 |
| SAP_PI597965 | 77.28 | 6.74 | 3.53 | 4.01 | 3.94 | 60.99 | 74.48 | 75.85 |
| SAP_PI597966 | 70.88 | 6.97 | 2.14 | 2.19 | 1.94 | 34.37 | 32.93 | 41.78 |
| SAP_PI597967 | 80.34 | 6.99 | 3.04 | 3.33 | 3.63 | 50.77 | 56.25 | 66.10 |
| SAP_PI597968 | 85.71 | 6.82 | 2.26 | 2.69 | 2.81 | 55.61 | 69.65 | 71.07 |
| SAP_PI597971 | 77.44 | 6.47 | 2.34 | 2.82 | 3.19 | 27.48 | 38.23 | 46.79 |
| SAP_PI597972 | 80.17 | 6.34 | 2.65 | 2.93 | 3.32 | 59.86 | 72.77 | 82.11 |
| SAP_PI597973 | 87.11 | 6.62 | 2.50 | 2.59 | 2.79 | 54.08 | 78.43 | 86.63 |
| SAP_PI597976 | 79.82 | 6.41 | 2.79 | 3.05 | 3.06 | 36.18 | 46.36 | 56.74 |
| SAP_PI597980 | 72.96 | 6.99 | 2.51 | 2.39 | 2.54 | 41.52 | 54.97 | 56.17 |
| SAP_PI597982 | 80.67 | 6.87 | 2.63 | 3.35 | 3.43 | 28.73 | 46.96 | 60.81 |
| SAP_PI598069 | 78.27 | 6.10 | 2.25 | 2.43 | 2.77 | 36.78 | 48.77 | 74.57 |
| SAP_PI601816 | 92.11 | 6.44 | 2.03 | 2.23 | 2.78 | 52.90 | 51.39 | 54.29 |
| SAP_PI607931 | 83.83 | 6.84 | 2.60 | 3.28 | 3.39 | 39.90 | 65.77 | 74.33 |
| SAP_PI609456 | 81.04 | 6.38 | 2.30 | 2.52 | 2.54 | 44.17 | 47.76 | 59.60 |
| SAP_PI613536 | 73.95 | 7.29 | 2.27 | 2.42 | 2.64 | 40.08 | 48.24 | 50.83 |
| SAP_PI629034 | 79.80 | 6.05 | 2.39 | 3.19 | 3.68 | 68.82 | 98.29 | 99.32 |
| SAP_PI629040 | 79.37 | 7.03 | 3.01 | 3.75 | 4.12 | 62.36 | 80.09 | 90.08 |
| SAP_PI629059 | 82.79 | 7.07 | 3.13 | 3.34 | 3.61 | 74.72 | 96.03 | 100.43 |
| SAP_PI641849 | 74.10 | 5.72 | 2.76 | 3.19 | 3.28 | 27.99 | 52.10 | 59.58 |
| SAP_PI641874 | 86.89 | 6.71 | 2.36 | 2.56 | 2.74 | 36.53 | 42.74 | 51.87 |
| SAP_PI642992 | 66.06 | 6.27 | 2.69 | 3.14 | 3.39 | 54.82 | 63.92 | 71.49 |
| SAP_PI642998 | 88.68 | 6.51 | 1.72 | 2.37 | 2.46 | 21.31 | 25.88 | 26.11 |
| SAP_PI65121 | 80.31 | 6.96 | 3.15 | 3.55 | 3.91 | 65.05 | 79.27 | 85.52 |
| SAP_PI651492 | 76.46 | 6.68 | 1.89 | 2.55 | 2.61 | 8.07 | 24.25 | 34.92 |
| SAP_PI651496 | 73.34 | 6.90 | 1.89 | 2.27 | 2.26 | 55.85 | 44.79 | 48.11 |
| SAP_PI655970 | 80.87 | 6.66 | 2.03 | 1.58 | 1.85 | 17.28 | 62.16 | 67.17 |
| SAP_PI655971 | 79.14 | 6.65 | 2.82 | 3.29 | 3.39 | 41.26 | 65.27 | 67.24 |
| SAP_PI655972 | 77.19 | 7.03 | 2.21 | 2.82 | 3.08 | 28.14 | 35.48 | 51.79 |
| SAP_PI655973 | 107.69 | 6.16 | 2.55 | 3.35 | 3.92 | 89.00 | 102.84 | 102.75 |
| SAP_PI655974 | 84.42 | 7.03 | 1.97 | 2.53 | 2.52 | 32.50 | 51.48 | 52.91 |
| SAP_PI655975 | 78.42 | 7.34 | 2.41 | 2.99 | 3.15 | 23.07 | 44.34 | 53.62 |
| SAP_PI655976 | 82.56 | 6.14 | 3.34 | 3.93 | 4.20 | 71.01 | 90.09 | 94.54 |
| SAP_PI655977 | 72.45 | 6.98 | 2.06 | 2.37 | 2.35 | 11.13 | 27.69 | 32.24 |
| SAP_PI655978 | 73.75 | 6.33 | 2.57 | 2.63 | 2.50 | 53.03 | 79.57 | 92.13 |
| SAP_PI655980 | 75.38 | 7.25 | 2.31 | 3.00 | 2.86 | 25.57 | 41.40 | 47.30 |
| SAP_PI655981 | 74.11 | 6.22 | 2.62 | 3.16 | 3.16 | 19.24 | 29.30 | 30.07 |
| SAP_PI655982 | 78.63 | 6.41 | 2.38 | 2.76 | 2.49 | 53.21 | 69.27 | 90.56 |
| SAP_PI655983 | 88.49 | 6.96 | 1.94 | 2.46 | 2.40 | 15.70 | 28.57 | 35.46 |
| SAP_PI655985 | 84.28 | 7.42 | 2.88 | 3.17 | 3.22 | 53.86 | 78.29 | 85.58 |
| SAP_PI655986 | 78.07 | 7.17 | 2.67 | 3.20 | 3.35 | 43.42 | 73.43 | 84.61 |
| SAP_PI655987 | 76.94 | 6.05 | 2.23 | 2.69 | 2.71 | 43.04 | 49.82 | 52.42 |
| SAP_PI655988 | 75.02 | 6.01 | 2.15 | 2.48 | 2.51 | 44.71 | 74.49 | 77.00 |
| SAP_PI655989 | 79.00 | 7.32 | 1.82 | 2.37 | 2.38 | 26.08 | 63.90 | 73.57 |
| SAP_PI655990 | 86.83 | 6.53 | 2.23 | 2.43 | 2.42 | 64.60 | 73.29 | 62.22 |
| SAP_PI655991 | 80.39 | 6.75 | 2.35 | 2.84 | 2.85 | 30.51 | 47.40 | 67.84 |
| SAP_PI655992 | 78.73 | 7.16 | 2.15 | 2.66 | 2.67 | 24.56 | 47.55 | 39.04 |
| SAP_PI655993 | 78.86 | 7.07 | 2.67 | 3.00 | 3.08 | 38.71 | 54.38 | 65.93 |
| SAP_PI655994 | 82.01 | 6.51 | 3.11 | 3.81 | 4.05 | 56.13 | 79.05 | 86.29 |
| SAP_PI655995 | 99.72 | 6.90 | 2.03 | 2.46 | 2.57 | 8.99 | 28.00 | 44.94 |
| SAP_PI655997 | 86.37 | 6.71 | 3.48 | 3.77 | 4.27 | 78.48 | 92.10 | 90.71 |
| SAP_PI655998 | 80.72 | 6.86 | 1.92 | 2.49 | 2.50 | 25.69 | 52.41 | 54.03 |
| SAP_PI655999 | 89.28 | 6.08 | 1.35 | 2.43 | 2.45 | 13.53 | 23.52 | 31.53 |
| SAP_PI656000 | 76.93 | 6.97 | 2.11 | 2.49 | 2.49 | 23.25 | 39.04 | 47.88 |
| SAP_PI656001 | 87.68 | 7.02 | 3.20 | 3.25 | 3.65 | 53.59 | 84.21 | 96.51 |
| SAP_PI656003 | 81.63 | 7.17 | 2.20 | 2.78 | 2.76 | 19.74 | 45.62 | 53.74 |
| SAP_PI656004 | 80.70 | 6.91 | 2.14 | 2.85 | 2.85 | 46.71 | 72.46 | 76.14 |
| SAP_PI656010 | 83.43 | 7.12 | 2.17 | 2.78 | 3.06 | 42.82 | 79.58 | 88.37 |
| SAP_PI656011 | 88.99 | 6.36 | 1.99 | 2.50 | 2.52 | 9.37 | 75.62 | 77.83 |
| SAP_PI656012 | 74.19 | 6.39 | 3.02 | 3.21 | 3.51 | 66.99 | 76.69 | 96.63 |
| SAP_PI656013 | 87.48 | 7.21 | NA | 2.20 | 2.10 | NA | NA | NA |
| SAP_PI656014 | 87.66 | 6.16 | 3.16 | 2.61 | 2.59 | 74.21 | 65.31 | 73.80 |
| SAP_PI656015 | 86.73 | 6.24 | 2.12 | 2.66 | 2.75 | 72.55 | 65.38 | 66.37 |
| SAP_PI656016 | 84.93 | 6.53 | 2.92 | 3.52 | 3.53 | 85.33 | 97.54 | 98.20 |
| SAP_PI656017 | 63.82 | 5.95 | NA | NA | 3.76 | NA | NA | 72.56 |
| SAP_PI656018 | 84.51 | 6.95 | 2.72 | 3.15 | 3.23 | 47.24 | 63.28 | 85.24 |
| SAP_PI656019 | 78.77 | 6.70 | 3.08 | 3.53 | 3.93 | 61.94 | 71.53 | 83.08 |
| SAP_PI656022 | NA | NA | NA | NA | 3.65 | NA | NA | 65.78 |
| SAP_PI656023 | 75.39 | 6.48 | 2.26 | 2.88 | 3.00 | 21.44 | 45.97 | 52.73 |
| SAP_PI656025 | 78.69 | 6.81 | 2.58 | 2.60 | 2.58 | 32.61 | 64.78 | 65.25 |
| SAP_PI656026 | 86.06 | 7.09 | 2.34 | 2.80 | 2.77 | 25.73 | 47.85 | 60.25 |
| SAP_PI656027 | 84.87 | 7.08 | NA | NA | 4.55 | NA | NA | 65.75 |
| SAP_PI656028 | 76.90 | 6.65 | 2.19 | 2.50 | 2.53 | 23.93 | 73.04 | 77.11 |
| SAP_PI656029 | 85.28 | 7.03 | 2.43 | 2.90 | 2.93 | 42.43 | 60.16 | 72.00 |
| SAP_PI656030 | 79.87 | 6.70 | 2.40 | 2.93 | 3.28 | 50.67 | 68.23 | 79.59 |
| SAP_PI656031 | 83.77 | 6.79 | 2.29 | 2.59 | 2.58 | 41.93 | 56.76 | 70.68 |
| SAP_PI656032 | 77.22 | 6.51 | 1.92 | 2.28 | 2.16 | 15.02 | 34.68 | 40.66 |
| SAP_PI656033 | 88.07 | 6.27 | NA | NA | NA | NA | NA | NA |
| SAP_PI656034 | 78.48 | 6.27 | 1.72 | 2.04 | 2.08 | 11.50 | 51.27 | 55.54 |
| SAP_PI656035 | 72.13 | 6.71 | 1.83 | 2.20 | 2.19 | 16.71 | 20.92 | 22.19 |
| SAP_PI656036 | 76.27 | 7.13 | 2.16 | 2.49 | 2.38 | 35.94 | 38.13 | 47.49 |
| SAP_PI656037 | 74.42 | 6.90 | 1.60 | 1.93 | 1.95 | 68.23 | 85.98 | 75.52 |
| SAP_PI656038 | 77.55 | 6.41 | 2.03 | 2.44 | 2.45 | 18.63 | 37.09 | 41.89 |
| SAP_PI656041 | 79.65 | 6.95 | 2.58 | 2.78 | 2.74 | 60.49 | 69.76 | 80.13 |
| SAP_PI656043 | 86.65 | 6.38 | 1.92 | 1.99 | 1.69 | NA | NA | NA |
| SAP_PI656044 | 84.73 | 7.38 | 2.00 | 2.19 | 2.15 | 25.26 | 47.22 | 47.14 |
| SAP_PI656046 | 82.36 | 6.45 | 2.40 | 2.68 | 2.82 | 36.32 | 68.82 | 80.36 |
| SAP_PI656047 | NA | NA | NA | NA | NA | NA | NA | NA |
| SAP_PI656048 | 78.58 | 6.65 | 2.39 | 2.21 | 2.23 | 29.23 | 55.91 | 73.77 |
| SAP_PI656049 | 78.52 | 6.76 | 2.98 | 3.53 | 3.57 | 41.65 | 73.00 | 78.34 |
| SAP_PI656050 | 76.93 | 6.52 | 2.03 | 2.42 | 2.46 | 12.82 | 20.72 | 32.04 |
| SAP_PI656051 | 87.92 | 7.22 | 1.95 | 2.77 | 2.77 | 37.72 | 75.49 | 79.80 |
| SAP_PI656052 | 80.29 | 7.46 | 2.81 | 3.36 | 3.25 | 39.47 | 53.12 | 60.30 |
| SAP_PI656053 | 87.20 | 7.40 | 1.92 | 2.40 | 2.53 | 26.08 | 64.31 | 68.95 |
| SAP_PI656055 | 92.15 | 6.45 | 2.31 | 2.55 | 2.56 | 33.57 | 66.95 | 68.96 |
| SAP_PI656056 | 84.88 | 6.65 | 2.49 | 3.72 | 4.17 | 59.51 | 69.15 | 81.68 |
| SAP_PI656058 | 89.88 | 6.60 | 2.49 | 2.52 | 2.46 | 46.79 | 70.67 | 75.62 |
| SAP_PI656059 | 87.24 | 6.97 | NA | NA | 1.71 | NA | NA | 99.60 |
| SAP_PI656062 | 94.76 | 7.09 | 2.59 | 2.56 | 2.58 | 21.05 | 51.54 | 50.61 |
| SAP_PI656064 | 90.63 | 6.62 | 1.45 | 2.35 | 2.54 | NA | 27.76 | 38.53 |
| SAP_PI656065 | NA | NA | NA | NA | 3.25 | NA | NA | 58.65 |
| SAP_PI656066 | 75.02 | 6.74 | 2.21 | 2.46 | 2.45 | 39.21 | 67.77 | 55.54 |
| SAP_PI656067 | 80.19 | 6.61 | 2.41 | 2.79 | 2.86 | 45.16 | 72.67 | 79.07 |
| SAP_PI656068 | 89.48 | 6.98 | NA | NA | NA | NA | NA | NA |
| SAP_PI656070 | 78.31 | 7.32 | 2.40 | 2.44 | 2.51 | 40.24 | 57.83 | 65.01 |
| SAP_PI656071 | 72.43 | 7.04 | 2.26 | 2.78 | 2.77 | 42.50 | 53.31 | 63.55 |
| SAP_PI656072 | 94.25 | 6.46 | 1.96 | 2.33 | 2.35 | 28.31 | 44.59 | 47.73 |
| SAP_PI656074 | 80.78 | 6.44 | 1.89 | 2.79 | 2.69 | 35.72 | 57.33 | 62.96 |
| SAP_PI656075 | 77.83 | 7.46 | 2.45 | 2.75 | 2.93 | 57.61 | 60.02 | 74.18 |
| SAP_PI656076 | 84.40 | 6.45 | 2.13 | 2.21 | 2.34 | 41.15 | 56.97 | 64.00 |
| SAP_PI656077 | 76.66 | 6.27 | 3.56 | 4.28 | 4.32 | 72.46 | 91.84 | 92.79 |
| SAP_PI656078 | 78.70 | 5.90 | 2.31 | 2.80 | 2.76 | 40.87 | 46.50 | 49.03 |
| SAP_PI656079 | 81.07 | 5.89 | 2.35 | 2.79 | 2.85 | 28.70 | 42.63 | 46.23 |
| SAP_PI656080 | 78.68 | 6.21 | 2.40 | 2.57 | 2.83 | 25.77 | 42.15 | 48.66 |
| SAP_PI656081 | 80.92 | 6.60 | 2.73 | 2.93 | 3.05 | 34.55 | 66.65 | 84.42 |
| SAP_PI656082 | 92.38 | 6.87 | 2.22 | 2.54 | 2.66 | 33.42 | 53.18 | 70.08 |
| SAP_PI656083 | 93.41 | 5.98 | 2.31 | 2.55 | 2.55 | 57.20 | 62.96 | 69.80 |
| SAP_PI656086 | 91.46 | 6.82 | 2.43 | 3.09 | 3.39 | 48.22 | 71.67 | 72.14 |
| SAP_PI656087 | 81.91 | 6.26 | 2.27 | 3.10 | 3.59 | 36.17 | 73.50 | 96.85 |
| SAP_PI656088 | 95.48 | 5.98 | 2.37 | 2.85 | 2.87 | 36.59 | 80.56 | 81.56 |
| SAP_PI656089 | 85.49 | 6.34 | 3.02 | 3.49 | 3.46 | 55.20 | 84.63 | 87.73 |
| SAP_PI656090 | 75.37 | 6.59 | 2.69 | 2.79 | 2.86 | 13.40 | 25.67 | 38.74 |
| SAP_PI656091 | 89.76 | 6.67 | 2.21 | 2.48 | 2.88 | 32.49 | 55.42 | 80.31 |
| SAP_PI656092 | NA | NA | NA | 1.80 | 1.98 | NA | 100.94 | 96.49 |
| SAP_PI656093 | 75.58 | 6.55 | 2.64 | 3.21 | 3.26 | 40.74 | 69.86 | 71.75 |
| SAP_PI656094 | 78.12 | 7.21 | 2.14 | 2.33 | 2.41 | 26.42 | 38.77 | 42.72 |
| SAP_PI656095 | 82.13 | 6.56 | 3.18 | 3.59 | 3.94 | 70.03 | 80.91 | 81.43 |
| SAP_PI656096 | NA | 6.86 | NA | NA | 3.68 | NA | NA | 65.67 |
| SAP_PI656097 | 102.80 | 6.47 | 2.94 | 2.83 | 2.90 | 75.34 | 73.60 | 80.05 |
| SAP_PI656101 | 72.98 | 6.46 | 2.25 | 2.65 | 3.06 | 25.43 | 40.51 | 47.08 |
| SAP_PI656102 | 77.62 | 7.07 | 2.23 | 2.97 | 2.95 | 18.51 | 33.95 | 44.08 |
| SAP_PI656104 | 76.70 | 6.46 | 3.14 | 3.68 | 3.68 | 42.37 | 74.47 | 74.56 |
| SAP_PI656105 | 80.62 | 6.82 | 2.13 | 2.91 | 2.91 | 24.92 | 32.49 | 38.38 |
| SAP_PI656106 | 82.05 | 6.66 | 2.15 | 2.61 | 2.68 | 45.45 | 66.98 | 71.60 |
| SAP_PI656107 | 90.12 | 6.74 | 2.41 | 3.19 | 3.47 | 37.70 | 78.81 | 93.47 |
| SAP_PI656108 | 76.97 | 7.23 | 3.37 | 3.71 | 3.86 | 54.48 | 65.14 | 77.07 |
| SAP_PI656109 | 74.18 | 6.81 | 3.61 | 3.87 | 3.95 | 61.22 | 75.58 | 82.27 |
| SAP_PI656110 | 89.63 | 6.93 | 2.53 | 3.05 | 3.16 | 25.84 | 43.74 | 48.01 |
| SAP_PI656111 | 87.05 | 6.90 | 3.46 | 3.68 | 3.82 | 63.20 | 81.80 | 87.49 |
| SAP_PI656112 | 76.63 | 6.61 | 2.97 | 3.26 | 3.65 | 54.00 | 70.41 | 88.72 |
| SAP_PI656114 | 80.71 | 6.57 | 2.97 | 3.69 | 3.95 | 55.92 | 87.36 | 95.66 |
| SAP_PI656116 | 81.93 | 6.47 | 3.24 | 3.96 | 4.02 | 74.83 | 85.29 | 96.27 |
| SAP_PI656118 | 83.89 | 7.46 | 2.63 | 3.24 | 3.58 | 39.32 | 68.61 | 71.57 |
| SAP_PI656119 | 79.26 | 6.50 | 3.28 | 3.72 | 3.76 | 68.96 | 87.62 | 96.59 |
| SAP_PI656120 | 74.74 | 6.63 | 3.12 | 2.70 | 2.69 | 118.64 | 107.33 | 102.42 |
| SAP_PI656121 | 77.06 | 6.41 | 2.58 | 2.88 | 2.89 | 25.42 | 41.20 | 48.26 |

WAP - weeks after planting, DTF - days to 50% flowering and PQ - pollen quantity
